# Prediction of Mortality in Very Premature Infants: A Systematic Review of Prediction Models

**DOI:** 10.1371/journal.pone.0023441

**Published:** 2011-09-08

**Authors:** Stephanie Medlock, Anita C. J. Ravelli, Pieter Tamminga, Ben W. M. Mol, Ameen Abu-Hanna

**Affiliations:** 1 Department of Medical Informatics, Academic Medical Center, University of Amsterdam, Amsterdam, The Netherlands; 2 Department of Neonatology, Academic Medical Center, University of Amsterdam, Amsterdam, The Netherlands; 3 Department of Obstetrics and Gynecology, Academic Medical Center, University of Amsterdam, Amsterdam, The Netherlands; Hôpital Robert Debré, France

## Abstract

**Context:**

Being born very preterm is associated with elevated risk for neonatal mortality. The aim of this review is to give an overview of prediction models for mortality in very premature infants, assess their quality, identify important predictor variables, and provide recommendations for development of future models.

**Methods:**

Studies were included which reported the predictive performance of a model for mortality in a very preterm or very low birth weight population, and classified as development, validation, or impact studies. For each development study, we recorded the population, variables, aim, predictive performance of the model, and the number of times each model had been validated. Reporting quality criteria and minimum methodological criteria were established and assessed for development studies.

**Results:**

We identified 41 development studies and 18 validation studies. In addition to gestational age and birth weight, eight variables frequently predicted survival: being of average size for gestational age, female gender, non-white ethnicity, absence of serious congenital malformations, use of antenatal steroids, higher 5-minute Apgar score, normal temperature on admission, and better respiratory status. Twelve studies met our methodological criteria, three of which have been externally validated. Low reporting scores were seen in reporting of performance measures, internal and external validation, and handling of missing data.

**Conclusions:**

Multivariate models can predict mortality better than birth weight or gestational age alone in very preterm infants. There are validated prediction models for classification and case-mix adjustment. Additional research is needed in validation and impact studies of existing models, and in prediction of mortality in the clinically important subgroup of infants where age and weight alone give only an equivocal prognosis.

## Introduction

Preterm birth is the single largest contributer to neonatal mortality in developed countries [1]. Mortality risks are especially high in the very low gestational age (VLGA, less than 32 weeks) and very low birth weight (VLBW, less than 1500 g) populations, and survival is even less certain in the ELGA/BW infants (extremely low gestational age/birth weight, less than 28 weeks and less than 1000 g). Physicians and parents are faced with difficult decisions at every stage of care: deciding if and when to intervene with Cesarean section, whether resuscitation should be attempted and mechanical ventilation or other treatments should be initiated, and whether and when treatment should be withdrawn. An accurate prognosis could help ease these difficult decisions. Consequently, prediction of mortality in very preterm infants is of the utmost importance.

Multivariate prediction models combine individual patient characteristics to predict a diagnostic or prognostic outcome [2]. Prediction models can be used to make a prognosis for an individual patient, or to stratify patients in the arms of a clinical trial, or for case-mix adjustment when comparing two populations in quality improvement efforts [3].

A clinician or researcher who wishes to use a prediction model in their country or setting can choose between using an existing model, recalibrating and validating an existing model (determining if it performs well in the local population), or creating a new model (if datasets are available). While there are many existing models for predicting mortality in very preterm infants, there is no overview of these models and their quality.

The objective of this review is to systematically review models for the prediction of mortality in very premature infants, identify promising variables which are frequently significant in multivariate models, and assess the quality of these models in order to provide recommendations for future research.

## Methods

### Study selection

The Cochrane library of systematic reviews was searched and no reviews on this subject were found. The MedLine database was searched for all articles indexed up to May 2010. The search followed the general form: prediction model AND preterm AND infant AND mortality. A complete list of search terms is given in Table S1.

The articles were divided among three reviewers (SM, AR, and AAH) such that two independently inspected each title and abstract, and marked those relevant for inclusion. Disagreement was resolved by discussion with all three reviewers. The full texts of the selected articles were then reviewed for final inclusion and divided into studies describing development of new models, studies which validated an existing model, and studies of the model's impact on clinical decision making. The references of included studies were searched manually to identify additional articles, and articles citing the included papers were identified and checked. The search was limited to articles available in English, excluding letters and comments.

### Inclusion criteria

The inclusion criteria were:

- The study should report on a population of live-born infants born at less than 32 weeks gestational age and/or less than 1500 g birth weight. Prediction models for a gestational age- or birth weight-specific subpopulation were included. Studies which used a slightly broader definition of VLGA/BW or ELGA/BW were included. Prediction models for a subpopulation with a specific disease or condition (for example, VLBW infants with necrotizing enterocolitis) were excluded. Prediction models for a general neonatal intensive care unit (NICU) population were excluded unless they reported performance separately for VLGA/BW infants.
- The outcome which the prediction model predicts is neonatal mortality or survival, or a combined outcome which includes mortality or survival. Studies which report on both stillbirth and neonatal mortality are included.
- The purpose of the model is to predict the probability of survival, rather than to investigate a single specific risk factor.
- The study must report at least one measure of the predictive performance of a multivariate model (discrimination, calibration, or accuracy).

### Data collection and analysis

Included studies were classified as development, validation, or impact studies. Development studies describe and assess the performance of a prediction model which was not previously published, or modified a previous model by adding or removing variables. Validation studies assess the performance of a previously published prediction model in a new population, or recalibrate a previous model by mathematically adjusting the model without changing the variables used. Impact studies assess the effect a prediction model has on clinical decisions. Validation studies were used only to count the number of times a model described in a development study had been validated. From each development study, data were collected on the study population, the prediction model or models developed, the performance and validation of the prediction model, and the quality of the study, using a structured data collection form.

#### Population

The population is defined by the range of gestational ages or birth weights, the setting, study year, and (postpartum) age of the infants at inclusion in the study. Maturity (gestational age and/or birth weight) is a strong predictor for mortality, and thus models with a wide range of gestational ages and birth weights are expected to have better performance measures. The study year and setting indicate whether treatments such as surfactant and antenatal steroids were available. The age of the infant at inclusion (for example at the onset of labor, at live birth, or at 24 hours postpartum) determines how long the infant needed to survive before being included in the study population, and thus strongly influences the composition of the study population. This is due to the substantial mortality in each of the early stages of life: intrapartum, in the delivery room, and in the first hours of NICU care.

Since the performance of prediction models can only be compared between similar populations, populations were classified as VLGA/BW or ELGA/BW, developed or developing county, pre- or post- surfactant era, and by mortality rate for the purpose of comparing model performance. Mortality rates were considered comparable if the absolute difference was ≤10%. Studies were classified as pre- or post- surfactant based on the authors' report of surfactant use. In studies where surfactant use was not reported, surfactant was assumed to be in routine use after 1995 in developed countries.

#### Prediction models

The time of prediction and type of prediction model were recorded. The time of prediction is the time at which the prediction model can be used, e.g. models for antenatal prediction versus prediction after NICU data is available. The prediction model type was categorized as logistic regression, neural network, classification tree, or other [2]. If reported, the model (equation or score) was recorded.

#### Variables

The outcome variable and input variables were collected for each prediction model. The input variables are the potential predictors which were tested, both during model development and in the final model. Interaction terms and mathematical transformations were not considered new variables (e.g. age and age^2^ refer to the same input variable).

#### Performance measures

Performance measures (discrimination, calibration, and accuracy) and the range of probabilities given by the prediction model were recorded. Discrimination measures whether the prediction model gives higher probabilities to patients with the event (e.g. patients who die) as opposed to patients without the event, commonly measured by the AUC (Area Under the receiver operating characteristic Curve). Calibration measures whether the predicted probability is approximately correct (e.g. when the model predicts a 30% chance of survival, then about 30% of patients are expected to survive). Accuracy is a measure of how close, on average, a prediction for a patient is to his or her actual outcome. The prediction is usually a percent probability, but it can be categorized into 0 or 1 (survival or non-survival) based on a cutoff point. Measures of accuracy may combine aspects of discrimination and calibration.

Different measures are important for different uses of prediction models. The intended use of the prediction model was derived from the aim or conclusions of the study and classified as classification (e.g. classification into high and low risk), case-mix adjustment (e.g. for quality control or benchmarking), clinical decision-making, or other. The performance measures were considered appropriate for the intended purpose if they included: for classification, a measure of discrimination or accuracy; for case-mix adjustment, a measure of discrimination and a measure of calibration or accuracy; and for clinical decision-making, a measure of discrimination, calibration, and the distribution or range of probabilities given by the model [2]. The distribution or range is particularly relevant for clinical usefulness, for example a model which predicts a 10% probability of survival for some infants and 90% for others is likely to be more useful than a model which only gives predictions of 49% and 51%.

#### Validation

Validation assesses model performance. A performance measure can be calculated from the same population which the model was developed (“apparent” validation), but this tends to inflate performance due to leveraging on coincidental correlations in the development population. A separate population can be created statistically by sampling subpopulations (e.g. bootstrapping), or by reserving a separate sample of patients for validation. Internal validation tests performance in the same setting where the model is developed, and external validation tests in a different setting [4].

#### Quality assessment

A framework was developed for the purpose of assessing the quality of the prediction models and articles. The framework was synthesized from quality assessment frameworks for prediction models of pregnancy in subfertile couples [4] and for illness severity in adult ICU patients [5], and recommendations for developing good prediction models [2], [6]. The framework was divided into two sections: a reporting score which assessed the quality of the article and a methodological score which consists of a set of minimum criteria for developing a valid prediction model. Each item in the scores was rated as no (0 points), partly (1 point), or yes (2 points). There were 19 items in the reporting score for a total of 38 possible points, and 6 items in the methodological score for a total of 12 possible points.

## Results

### Search results

**Figure 1:**
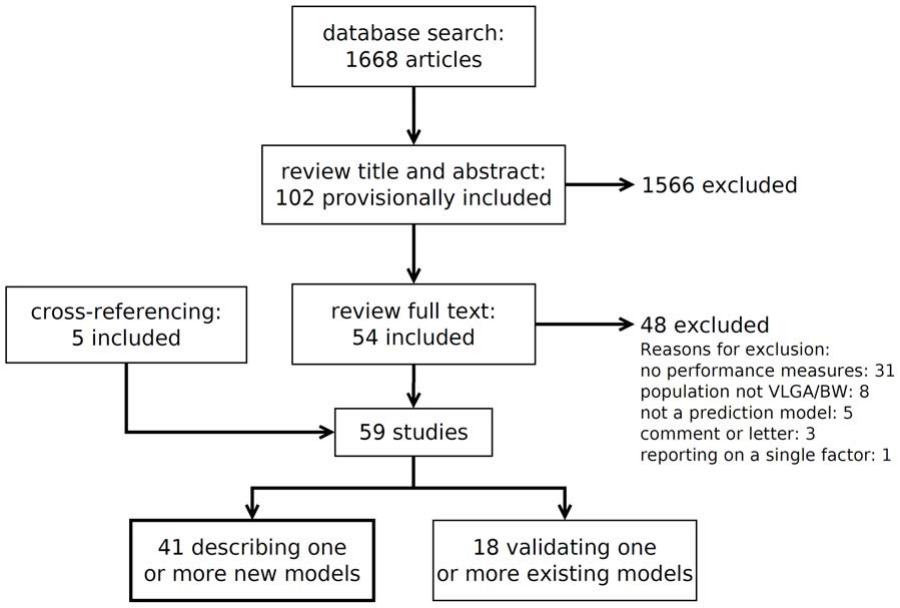
Results of search. This review focuses on the 41 development studies, which describe previously unpublished models predicting mortality.

The results of the search are summarized in Figure 1. The search resulted in 1668 articles, of which 102 were provisionally included on the basis of title and abstract. Review of the full text retained 54 articles for inclusion. Review of the references and cross-referencing of the included studies yielded a further 5 papers for inclusion, for a total of 59 studies. Of these, 41 described development of a previously unpublished model and 18 reported on validation of one or more previously published models. No impact studies were found. Of the 41 studies reporting on model development, 23 reported more than one model (range  = 1–10, median  = 2), resulting in 103 unique prediction models.

### Study data and analysis

Table S2 summarizes the population, aim, performance measures, validation, and quality of the 41 development studies. Further details of these 41 studies can be found in Table S3.

#### Populations

The populations of the included studies were heterogeneous. The studies included 28 which predicted outcome for a general VLGA/BW population [7]–[34] and 13 studies specific to ELGA/BW infants [35]–[47]. Of these 13 studies, 7 focused on the youngest and smallest of the ELGA/BW infants [36], [38], [39], [41], [44]–[46].

Studies dated from 1982–2010. The included studies represent populations from 21 countries, and seven of the included prediction models are specifically for use in low-resource or mixed-resource settings [16], [25], [27], [30]–[32], [34]. The number of VLGA/BW infants included in each of the studies ranged from 59 [41] to 12960 [15], and the mortality rate among these infants ranged from 6.8% [28] to 59.6% [37]. Infants were included in the study population at the onset of labor/decision to deliver in 3 studies, at live birth in 12 studies, on NICU admission or the start of mechanical ventilation in 13 studies, after survival of 1–24 hours in 5 studies, or survival for >1 day in 4 studies. The (postpartum) age of the infant at inclusion in the study was not reported in 6 studies.

#### Prediction models

**Figure 2:**
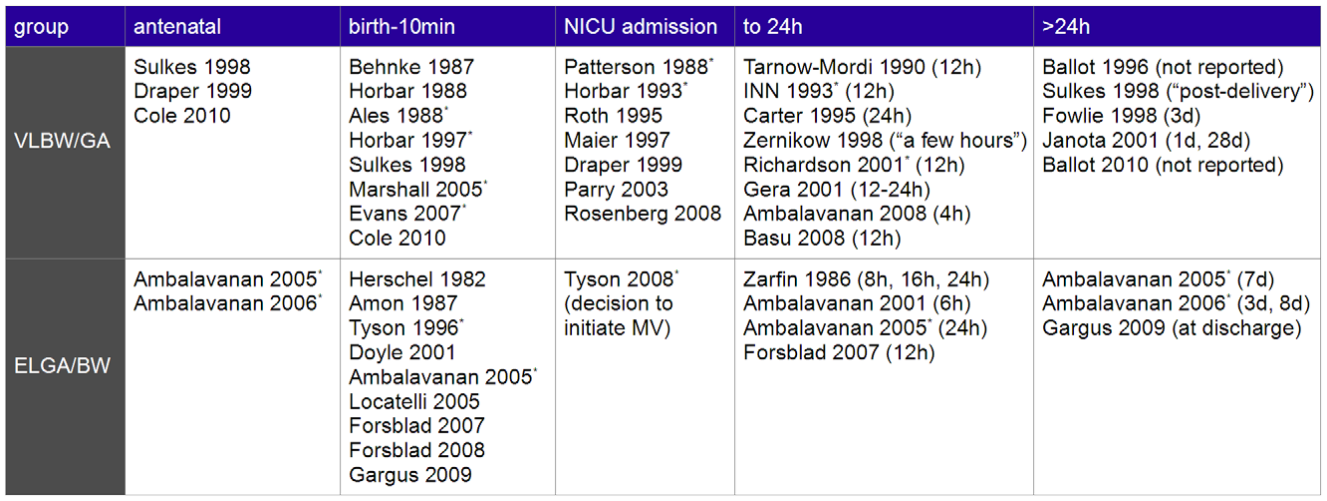
Classification of studies by the time at which a prediction can be made. Summary of prediction models by time of prediction. The time of prediction is the point in time where a prediction can be made using the model. A model for antenatal prediction was reported in 5 studies, for prediction at live birth in 17 studies, upon NICU admission in 8 studies, from the first day of life in 12 studies, and after the first day of life in 6 studies. Two studies do not specify when the data should be collected or when a prediction can be made. Studies marked with an asterisk met our criteria for methodological quality.

The times of prediction are summarized in Figure 2. Five studies include a prediction model which is intended to make an antenatal prediction, two of these for ELGA/BW infants. Seventeen studies developed a prediction model for making predictions using data available at birth, while a further eight use NICU admission data or data available at the start of mechanical ventilation. Twelve studies, including the Clinical Risk Index for Babies (CRIB [13]) and Score for Neonatal Acute Physiology-II (SNAP-II [23]), use data from the first few hours of life. Six studies include a prediction model which uses data available after the first day, and two studies do not specify when the data used to develop the prediction model were collected. Nine studies include multiple prediction models with different times of prediction [19], [22], [24], [33], [36], [42]–[44], [47]. Six of these compare different times of prediction [19], [33], [42]–[44], [47], all of which found that the addition of later data made little difference in the predictive performance of the models.

The majority of the included studies used a logistic regression model (35/41), with the others reporting on a classification tree [43], a log linear regression model [15], discriminant analysis [8], [35], [37], and a score based on expert opinion [24]. Four studies compared the performance of two different types of prediction models, three of which compared logistic regression to a neural network model. In one the neural network performed better (AUC  = 0.954 vs. 0.917, p = 0.002 by the univariate Z-score test of the difference in AUCs [21]), in the others the performance was approximately equal (AUC  = 0.87 in both models [40], and slightly better performance from the regression model in 3/5 scenarios [42]). The fourth study used both a CART (Classification And Regression Tree) model and logistic regression, but did not report the same outcome measures for the two models [29].

#### Dependent variable (outcome)

Most prediction models in this review (33) predict mortality, the remainder (8) predict survival. The observation period is 28 days (neonatal mortality) or the duration of hospital stay in 30/41 studies. Since late deaths are a very small fraction of the mortality in this population, it is expected that longer observation will not affect the choice of variables, their weight or significance in the final model, or the performance of the model.

Three studies indicated that infants who survived “against the odds” had a poor neurologic outcome [15], [36], [42]. However, models with a combined outcome of mortality and morbidity did not show better performance than models predicting mortality alone in studies which compared the two [16], [29], [39], [45]–[47].

#### Independent variables (predictors)

The 41 included studies investigated a total of 254 different input variables. A table showing categories of variables included in each study is given in Table S4. A complete list of the exact variables tested and their univariate and multivariate significance is available from the authors upon request.

In addition to higher gestational age and birth weight, 7 input variables were tested in 10 or more studies and were frequently found to predict improved survival in multivariate models: being of average size for gestational age (10/22 studies); female gender (18/30 studies); non-white ethnicity (9/20 studies); use of antenatal steroids (11/20 studies); higher Apgar score (18/26 studies); normal temperature on admission (5/12 studies); and a measure of respiratory status such as blood gas (11/16 studies), FiO_2_ (6/7 studies), or clinical respiratory function (9/19 studies).

Congenital malformation is often an exclusion criterion rather than an input variable and therefore was not an input variable in many studies, but was often significant when tested (4/8 studies). Multiple gestation showed an inconsistent effect. It was included in the final model in 8/20 studies, but showed a protective effect for singleton infants in 6 studies and multiple infants in 2 studies. Hospital care factors were variably significant, such as inborn/outborn status (3/10 studies) and hospital of birth (4/8 studies).

A number of input variables were frequently tested, but usually did not remain significant in the final models. Such variables include: maternal health risks (0/10 studies, except hypertension/preeclampsia which had a protective effect in 4/11 studies), maternal age (2/12 studies), premature rupture of membranes (1/10 studies), presentation (0/10), mode of delivery (2/25 studies), and infant morbidities (4/17 studies, with the exception of seizures (3/6 studies) and shock (2/2 studies)).

#### Performance measures

A summary of the reported performance measures is given in Table 1. The most common measure reported was the p-value of the Hosmer-Lemeshow statistics (19/41 studies), followed by the AUC (18/41 studies). Performance measures for the 103 models and the equations or scores used to make a prediction are provided in Table S3. Performance measures which were reported in ≥3 studies with a similar population are reported in Table 2. No more than four studies reported the same measure for any patient group, which was insufficient for meta-analysis.

**Table 1:** Summary of performance measures reported in the 41 development studies.

	measure	n studies	lowest	highest	mean	median
discrimination	AUC	18/41	0.698 [32]	0.954 [21]	0.8583	0.87
calibration	H-L p	19/41	<0.01 [42]	0.99 [18]	0.5757	0.6255
accuracy	% correct / accuracy	9/41	0.62 [43]	0.883 [14]	0.7857	0.79
	PPV	14/41	0.44 [18]	0.84 [27]	0.703	0.75
	NPV	15/41	0.522 [32]	0.94 [42]	0.7986	0.81
	sensitivity	10/41	0.17 [25]	0.95 [7]	0.6474	0.72
	specificity	13/41	0.32% [29]	1.00 [25]	0.7848	0.85
discrimination, calibration, accuracy	R2	4/41	0.30 [17]	0.69 [41]	0.3588	0.39
	range	15/41	27–52% [43]	1–98% [33]	--	--

Overview of performance measures reported by ≥2 studies. At least one performance measure was required for inclusion; most studies reported more than one. A summary of measures per study is given in Table S2, and the measures reported for each model are in Table S3. The heterogeneity of the studies precludes direct comparison of model performance.

AUC  =  area under the curve; ideal  = 1.0 and chance  = 0.5.

R^2^  =  coefficient of determination; the proportion of variability that is accounted for by the model. ideal  =  1.0.

H-L p  =  Hosmer-Lemeshow p value; any non-significant value indicates acceptable calibration.

% correct; ideal  = 1.0 and chance ≈ %survival^2^.

PPV  =  positive predictive value; ideal  = 1.0 and chance related to prevalence and cut-off.

NPV  =  negative predictive value; ideal  = 1.0 and chance related to prevalence and cut-off.

sensitivity; ideal  = 1.0 and chance related to prevalence.

specificity; ideal  = 1.0 and chance related to prevalence.

range  =  the range of probabilities generated by the model.

Note that performance measures of unvalidated models may be overestimated.

**Table 2:** Comparison of predictive performance of models as reported in the development studies.

VLGA/BW, post-surfactant, developed countries, AUC			
	age/weight range	mortality rate	performance
Zernikow 1998	<1500 g or <32 w	8.3%	AUC = 0.954 (SD 0.015)*
Parry 2003	< = 32 w	7.9%	AUC = 0.92 (SE 0.01)
Evans 2007	<1500 g or <32 w	6.8%	AUC = 0.83
**ELGA/BW, post-surfactant, developed countries, AUC**			
Tyson 1996	501–800 g	33.5%	AUC = 0.76
Ambalavanan 2001	<1000 g	34%	AUC = 0.87 (SE 0.03)*
Ambalavanan 2005	401–1000 g	35%	AUC = 0.854 (SE 0.004)*
Tyson 2008	401–1000 g and 22–25 w	42%	AUC = 0.753 (95% CI 0.737–0.769)
**ELGA/BW, post-surfactant, developed countries, R^2^**			
Ambalavanan 2001	<1000 g	34%	R^2^ = 0.36
Locatelli 2005	<750 g and <34 w	49.2%	R^2^ = 0.69†
Gargus 2009	401–1000 g	34.4%	R^2^ = 0.4175†

Studies which reported the same outcome measure were considered for comparison. Since the performance is only comparable in similar populations, the study populations were compared on whether the population was VLGA/BW or ELGA/BW, from a developed or developing country, from the pre- or post- surfactant era, and the reported mortality rate (an absolute difference of ≤10% was considered comparable). Only three categories contained more than two studies. All performance measures for each prediction model are given in Table S3.

*multiple models, using best performance of those reported.

† performance assessed on development set, may be overestimated.

Studies which directly compared the performance of their model to birth weight and/or gestational age (12 studies [7], [12], [13], [20], [21], [25]–[28], [32], [34], [45]) showed that the multivariate model performed better, with the exception of one study [25] which restricted the prediction model to information routinely available in a low-resource setting. Other prediction models developed in low-resource and mixed-resource settings do outperform birth weight [27], [32], [34]. Of the 12 studies comparing the model to birth weight and/or gestational age, the largest differences reported were an AUC of 0.70 for birth weight compared to an AUC of 0.89 for the multivariate model [20] and an AUC of 0.78 for birth weight compared to an AUC of 0.90 for the multivariate model (the CRIB-II score, p = 0.03) [13]. However, since one was in an ELGA/BW population and the other in a VLGA/BW population, it is not possible to say which model would perform better in any one population.

The authors' intended use of the prediction model was classified as classification (5 studies), case-mix adjustment (15 studies), clinical decision making (15 studies), or other purpose (e.g. identifying predictive variables, 12 studies). Six suggested more than one intended use (see Table S2).

#### Validation

Validation was performed for at least one prediction model in 17/41 studies, most commonly split-sample internal validation (14/17), with no validation in the original publication but later published separately for an additional 2 studies. Only 7 studies developed models which were ever validated in a later study, with 4 validated in more than one additional study (NICHD [12], CRIB [13], SNAP-II and SNAPPE-II [23], and CRIB-II [26]). Four of the development studies compared their model to a previously published model, thus providing validation of the other model: one comparing to the NICHD model [27], three to CRIB [25], [27] including the CRIB-II study [26], and one to CRIB-II [32]. In all cases the new model performed as well or better than its comparison, although in one study this performance measure was taken on the development sample [25], which will tend to overestimate performance. Four additional studies included the CRIB [16], [20], [44] or SNAPPE-II [31] score as an input variable.

#### Quality and quality assessment framework

**Figure 3:**
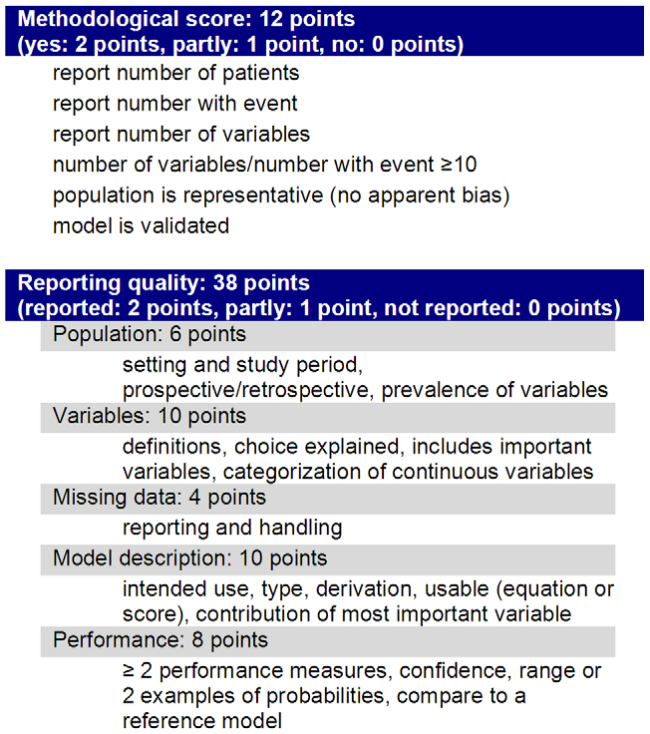
Summary of framework for assessing the quality of studies reporting on the development of a new prediction model. The quality framework consists of two parts, a methodological score with minimal criteria and a reporting score. The complete framework is available in the supporting text S1, along with an assessment of each included study.

A framework was developed to assess the quality of the studies, in terms of the quality of reporting and the quality of model development. A summary of the framework is given in Figure 3, the complete framework and the score of each study is given in the supporting text S1. One study [24] could not be evaluated using the framework, as it reported a score based on expert opinion rather than statistical methods.

Reporting scores ranged from 11 to 38 from a possible 38 points (median 28, interquartile range 24–33). One study scored 38/38 points on the reporting score [32]. In the other studies, common areas for improvement included reporting of the percentages of missing values and how they were handled, as only 17/40 studies reported both. A measure of confidence for at least one main performance measure was reported in 14/40 studies. Slightly more (22/40) reported more than one performance measure for at least one model, and 23/40 compared the performance of at least one model to birth weight, gestational age, or a reference model.

Methodological scores ranged from 7 to 12 from a possible 12 points (median 10, IQR 9-12), with 12 meeting all of our methodological criteria. The most common methodological problem was lack of validation, with less than half (17/40 studies) performing validation on a separate sample or using statistical validation techniques such as bootstrapping. In 9 studies there was some doubt about whether the population used to develop the model was representative, and in 6 the number of patients was not adequate to support the number of predicting variables.

The intended use of the model was classification in 2 studies [10], [13], case-mix adjustment in 7 [12], [13], [17], [23], [27], [28], [38], and clinical decision-making in 5 [8], [10], [38], [42], [45]. The performance measures reported were appropriate for classification and case-mix adjustment, but only one of the five reported all performance measures to assess the model for clinical decision making [10].

## Discussion

### Principal findings

This review identified 41 studies reporting on the development of a prediction model of mortality in very premature infants. There were 18 validation studies of existing prediction models and no impact studies. Nearly all studies found that a multivariate model predicted mortality better than birth weight or gestational age alone. In addition to gestational age and birth weight, eight variables frequently predicted survival: being of average size for gestational age, female gender, non-white ethnicity, absence of serious congenital malformations, use of antenatal steroids, higher 5-minute Apgar score, normal temperature on admission, and clinical or laboratory measures indicating better respiratory status. The included studies are heterogeneous in population and mortality rate. Twelve studies met our minimum methodological quality criteria, with eight for very premature infants and four for extremely premature infants. Room for improvement was found in the areas of reporting of model performance (reporting measures which are comparable between studies and are consistent with the stated objectives of the study), handling of missing values, and the use of efficient internal validation methods.

### Strengths and weaknesses

The primary strength of this study is in the systematic search strategy and systematic assessment of the quality and content of the included studies using a framework. There are of course limitations to any search strategy and it is possible that relevant articles may have been overlooked. Several choices have been made in this work which result in trade-offs. Our inclusion criteria intentionally excluded studies in a general NICU population, such as the study on the original SNAP score [48]. While this excludes some models with potential relevance, it ensures the relevance of models for our population of interest. We chose to limit our list of variables which were frequently found to be significant to those which had been considered as input variables in ten or more studies. The variables which we identified are thus more likely to be important, but this excludes variables which have not yet been extensively studied. We chose to limit our methodological score to minimal criteria, which results in less stratification than in the reporting score. However, the methodological score addresses the key question of whether the model was developed with sufficient rigor to form the basis for further research.

### Comparison to other studies

To our knowledge, this is the first systematic review of models for the prediction of mortality in very premature infants. A review was published in 2005 [3], summarizing 12 different scoring systems for neonatal morbidity at all birth weights and gestational ages. Six of those scoring systems, including CRIB, are also included in this review. In the year 2000 a comparison was published of the performance of 6 different scores, alone and in combination with one another and with birth weight [49], on a single VLBW population. The best model in that study was a combination of birth weight, birth weight squared, SNAP at 24-h, and Apgar <7 at 5 min with an AUC of 0.930 and a Hosmer-Lemeshow p value of 0.96, in contrast to birth weight alone which had an AUC of 0.869 and a Hosmer-Lemeshow p value of 0.0005.

### Implications

The primary audience of this systematic review are researchers who wish to further the state of knowledge in this area by developing, validating, or assessing the impact of prediction models. Clinicians should be aware that multivariate models including factors in addition to birth weight and gestational age predict survival better than these two factors alone, although generally only slightly better. The consistent survival advantage of female infants and infants of non-white ethnicity are of particular relevance to clinical decision-making.

#### Use of the prediction models

Prediction models have the potential to assist clinical decision making, case-mix adjustment, or classification. For clinical decision making, clinicians are often uncertain about resuscitation and initiating mechanical ventilation in infants at the edge of viability [50], where gestational age and birth weight alone give an equivocal prognosis. The gestational age and weight range of this group varies by year and by country, but is currently around 23 to 27 weeks in the developed world. Prediction models which are created with a smaller range of gestational ages and birth weights are likely to be more useful for clinical decisions in this group of infants, as the model will depend less on weight and age. No study in this review explicitly stated that the model was developed to assist with clinical decision making in the group of infants where clinicians feel uncertain in their clinical judgment, although the seven studies predicting mortality in the youngest and smallest ELGA/BW infants [36], [38], [39], [41], [44]–[46] may have been implicitly directed toward this goal. Clinical predictions based on the appearance of the infant at birth are often inaccurate [50], [51], and it is known that clinicians who over-predict death also tend to perform less resuscitation and other therapies, leading to the potential for a self-fulfilling prophecy [42]. The resuscitation policy and practices at the hospitals where these models were developed likely affected the outcome, and therefore possibly the prediction models. Resuscitation policy was reported in only 10 studies (see Table S3), although several others were multicenter studies which likely varied in resuscitation policy, and several specifically mentioned that resuscitation policy varied or was not known.

Of the aforementioned seven studies, two met our methodological quality criteria [38], [45]. Both are by the same research group and “take the perspective of a clinician deciding whether to initiate mechanical ventilation.” Although the authors do not report calibration or range in the more recent study, both models are good candidates for further work in this area. A good approach would be to determine which groups of infants pose a clinical dilemma for the participating hospitals, by observing or asking clinicians. Because preterm birth at very low gestational age is rather rare, international collaboration may be required in order to include sufficient numbers of infants to develop or validate a model. If a prediction model is found to have good performance on multiple measures, it can be externally validated and presented in a form which is usable for clinicians (for example, a wall chart or a computerized calculator, which can be very useful in counseling parents), and assessed in an impact study.

Models which make predictions for a general VLGA/BW population are most likely to be useful in classification, benchmarking, or case-mix adjustment. Scores such as CRIB [13] and SNAP-II [23] have been externally validated, but this review found six additional studies meeting our methodological criteria which predicted mortality for a general VLGA/BW population [8], [10], [12], [17], [27], [28]. These models could prove to be as good or better than CRIB or SNAP. General prediction models may also be recalibrated for a narrow range of maturity for clinical prediction, but performance is likely to be poorer as maturity itself is the largest predictor in most of these models. For the same reason, any prediction model for a narrow gestational age or birth weight range is likely to have poorer performance measures than a general prediction model, but may still make significantly better predictions than maturity or clinical intuition alone.

#### Variables

Users should be cautious when using prediction models which include variables that can be affected by treatment choices, and models which include subjective measures such as the Apgar score. Fetuses or infants who appear subjectively unlikely to survive may be treated differently than those who appear healthier, leading to differences in mortality. Subjective prognosis leading to different treatment decisions may explain why seizures predicted poor outcome (3/6 studies) but not intraventricular hemorrhage or periventricular leukomalacia (1/12 studies).

Whether an input variable remains in the final model depends on its ability to differentiate survivors from nonsurvivors, which depends in part on the prevalence of the variable in the population. For example, antenatal steroids are known to be beneficial, but appear in the final model in only 11/20 studies. In the other 9, antenatal steroids were either given routinely or rarely, thus the population was homogeneous in that respect. This may also explain why maturity was not significant in prediction models restricted to 23–24 [46] and 25-week [44] infants, and 1000–1500 g infants in a low-resource setting [16]. The importance of a variable in the model is also strongly influenced by the other variables which are included.

#### Study quality

This review identified three major areas for improvement of study quality: handling of missing data, validation, and reporting model performance. Simply omitting patients who have missing data can bias the population, resulting in a suboptimal model. Missing data can be handled by comparing populations with and without missing data to check for bias, imputing the missing values, or other appropriate techniques [2].

Most studies which performed an internal validation used a split sample. Future research can be improved by using resampling or bootstrapping techniques based on the whole study population, as they provide good evidence of validity without reducing sample size for model development [2]. Similarly, the most common performance measure reported in these studies is the Hosmer-Lemeshow p-value, a measure of calibration. This measure is strongly affected by the choice of equal-sized groups (C) or equal-interval groups (H), the choice of cut-points to create those groups, and sample size, and cannot be used by itself to compare models [52]. No one measure is ideal, and the best solution is to report two or more measures appropriate for the intended purpose of the model.

### Future work

For clinical use, external validation of a prediction model and impact studies are important indicators of its usefulness [53]. A summary of the scope and quality of external validation could be informative for future research. For clinicians and parents, predicting severe morbidity in total and on subgroups of diseases is equally important to decision-making. Although some models incorporated both outcomes, models predicting severe morbidity alone also warrant study. Meta-analyses focusing on specific prediction variables, such as the gender of the child and ethnicity of the child or mother, may help quantify the influence of those variables and the physiologic and social mechanisms which connect them to mortality.

Only four studies investigated the use of neural networks or classification trees [21], [29], [40], [42], three of which were by the same research team. Further investigation of these alternatives to logistic regression may be warranted [54]. Although some of the models predict mortality for the youngest and smallest ELGA/BW infants, no models were developed specifically for clinical prediction in infants where gestational age and birth weight give an equivocal prognosis. This may be due to the small number of infants who are born at this early age, but international collaboration could yield large enough data sets to develop and/or validate models for small ranges of gestational age and birth weight within this population. Models developed for the population at the tipping point of survival, where clinical judgment is most difficult, have the most potential to aid clinical decision making.

### Conclusion

This systematic review provides an overview of existing research in prediction of mortality in very preterm infants. Multivariate models generally predict mortality better than birth weight or gestational age alone. Eight common predictors were identified, including a consistent survival advantage for female infants and infants of non-white ethnicity. There are nine studies reporting validated prediction models for classification or case-mix adjustment which met our methodological criteria, though poor reporting of model performance precludes comparison of the predictive ability of these models. Future studies could be improved by reporting measures which are comparable between studies, reporting measures which are consistent with the stated objectives of the study, and comparing performance to a reference model. Better handling of missing data, better internal validation, and additional external validation studies are also needed. Five studies reported validated prediction models for clinical decision making, two of which focus on the youngest and smallest of premature infants. There are no studies of the impact of these models on clinical decision making. Future prediction models could focus on the narrow gestational age and weight ranges where clinicians feel uncertain in delivering a prognosis. Such models are most likely to help clinicians and parents in making the difficult decisions common to the care of very preterm infants.

## Supporting Information

Table S1 Terms used in database search. Search terms relating to the same concept were joined by “or.” Each concept group was joined by “and,” i.e. (predict- OR risk model OR AUC...) AND (preterm OR very-low-birth-weight...), etc.
(DOC)

Table S2 Summary of the 41 included studies reporting the development of a new prediction model. The 41 development studies were heterogeneous in population, aim, performance measures, validation, and quality. There were 28 studies for a general VLGA/BW population and 13 studies specific to ELGA/BW infants, dating from 1982–2010. The number of infants ranged from 59 to 12960 and the mortality rate from 6.8% to 59.6%. Further details of the included studies are given in Table S3.
(DOC)

Table S3 Detailed summary of the 41 included development studies.
(DOC)

Table S4 Summary of input variables used in the 41 development studies**.** A total of 241 different input variables were used in the 41 studies describing development of a new prediction model. This table summarizes the variables by category. A complete list of the exact input variables used in each study, and their univariate and multivariate significance, is available from the authors upon request.
(DOC)

Text S1 Framework for assessing quality of studies reporting the development of a prediction model, and scores of the 41 development studies.
(DOC)

Checklist S1 PRISMA checklist.
(DOC)

Diagram S1 PRISMA flow diagram.
(TIF)
