# Supplementary material for: Prediction of Mortality in Very Premature Infants: A Systematic Review of Prediction Models
Source: PLoS One. 2011 Sep 8;6(9):e23441. doi: 10.1371/journal.pone.0023441 (PMC3169543; doi:10.1371/journal.pone.0023441)
Supplement: Table S1 — Terms used in database search. Search terms relating to the same concept were joined by “or.” Each concept group was joined by “and,” i.e. (predict- OR risk model OR AUC...) AND (preterm OR very-low-birth-weight...), etc. (DOC) [file pone.0023441.s001.doc]

**Table S1: Terms used in database search**

|  | terms relating to: | joined by OR |
| --- | --- | --- |
|  | prediction models | predict-, risk model, AUC, area under the curve, receiver operating characteristic, ROC, discrimination, calibration, sensitivity and specificity, E/O, O/E, observed and expected, predictive value, prognostic model, risk categories, hosmer-lemeshow, risk score, or risk index |
| AND | prematurity | infant, premature (MESH term); infant, very low birth weight (MESH term); VLBW; ELBW; very low-birth-weight; extremely low-birth-weight; preterm; SGA; prematur-; low gestational age; to 1500 grams; or to 1000 grams |
| AND | mortality | survival, mortality, death, or viability |
| AND | infants | obstetric-, neonatal, infant, baby, midwife, or birth |

Search terms relating to the same concept were joined by "or." Each concept group was joined by "and," i.e. (predict- OR risk model OR AUC...) AND (preterm OR very-low-birth-weight...), etc.
