# Supplementary material for: Prediction of Mortality in Very Premature Infants: A Systematic Review of Prediction Models
Source: PLoS One. 2011 Sep 8;6(9):e23441. doi: 10.1371/journal.pone.0023441 (PMC3169543; doi:10.1371/journal.pone.0023441)
Supplement: Table S2 — Summary of the 41 included studies reporting the development of a new prediction model. The 41 development studies were heterogeneous in population, aim, performance measures, validation, and quality. There were 28 studies for a general VLGA/BW population and 13 studies specific to ELGA/BW infants, dating from 1982–2010. The number of infants ranged from 59 to 12960 and the mortality rate from 6.8% to 59.6%. Further details of the included studies are given in Table S3. (DOC) [file pone.0023441.s002.doc]

| **Table S2.** Summary of the 41 included studies reporting the development of a new prediction model | | | | | | | | | | | |
| --- | --- | --- | --- | --- | --- | --- | --- | --- | --- | --- | --- |
| name and  first author | year published  (study years) | country | GA/BW | number of patients  (mortality) | age at inclusion | outcome | aim of study | performance measures  & range | number of models | M: methodological and  R: reporting quality scores | validation |
| VLGA/BW (born at <32 weeks gestational age and/or <1500g birth weight) | | | | | | | | | | | |
| Behnke | 1987  (1974-80) | USA | VLBW  500-1800g | 748  (21.5%) | live birth | survival - 28d | other | *accuracy*: at 0.50; correct prediction, sens, spec, false pos, false neg | 2 | M: 9/12  R: 24/38 | none |
| Patterson | 1988  (1978-85) | UK | VLBW  <=1500g | 387  (22.2%) | NICU admission | mortality-discharge | clinical decisions | *accuracy*: at 0.50; % correct survivors and nonsurvivors  *range* | 1 | M: 12/12  R: 20/38 | split sample |
| Horbar | 1988  (1983-84) | US, CAN, UK | VLBW  701-1500g | 1776  (14.8%) | live birth | survival - 28d | classification | *calibration*: H-L | 2 | M: 10/12  R: 20/38 | none |
| Ales | 1988  (1986) | USA | VLBW  <=1500g | 158  (30%) | live birth | mortality- discharge | classification  clinical decisions | *discrimination*: Wilcoxon  *discrimination/calibration/accuracy*: R  *accuracy*: predictive accuracy  *range* | 1 | M: 12/12  R: 28/38 | temporally separate sample |
| Tarnow-Mordi | 1990  (1986-89) | UK | VLBW  <1500g, artificially ventilated | 262  (21.0%) | 12h | mortality- discharge | case-mix adjustment | *accuracy*: at 0.50; sens, spec, PPV, NPV, % correct classification | 4 | M: 9/12  R: 34/38 | mixed development and reserved sample |
| **NICHD**:  Horbar | 1993  (1987-89) | USA | VLBW  501-1500g | 3646  (24.7%) | live birth | mortality- discharge | case-mix adjustment | *discrimination:* AUC  *calibration:* goodness of fit  *accuracy*: at 50% and optimal (25%); sens, spec, PPV, NPV, % correct classification  *range* | 1 | M: 12/12  R: 34/38 | split sample  subsequent validation: 2 studies |
| **CRIB score**:  International Neonatal Network | 1993  (1988-90) | UK | VLGA/BW  <=1500g or <31w | 1198  (24.3%) | NICU admission | mortality- discharge | classification  case-mix adjustment | *discrimination:*AUC  *calibration:* H-L  *accuracy:*sens at 95% spec | 1 | M: 12/12  R: 35/38 | validated in different UK hospitals  subsequent validation: 17 studies[[1]](#footnote-2) |
| **NSI**:  Carter | 1995  (1980-83) | USA | VLBW  500-1800g | 309  (24.6%) | NICU admission | mortality- discharge | case-mix adjustment  clinical decisions | *accuracy*: at 0.50; sens, spec, % correct classification, ppv, npv | 1 | M: 7/12  R: 29/38 | none |
| Roth | 1995  (1980-83) | USA | VLBW  500-1500g | 12960  (19.2%) | NICU admission | mortality- discharge | clinical decisions | *calibration*: scaled deviance  *range* | 8 | M: 8/12  R: 25/38 | none |
| Ballot | 1996  (1992-94) | South Africa | VLGA/BW  <1501g or <31w | 231  (16%) | 12h | mortality- 28d | clinical decisions | *calibration*: goodness of fit | 1 | M: 10/12  R: 27/38 | none |
| Horbar | 1997  (1991-92) | North America, Europe, Asia, Australia | VLBW  501-1500g | 7672  (14.7%) | before NICU admission | mortality- 28d | case-mix adjustment | *discrimination*: AUC  *discrimination / calibration/accuracy:* R^2^  *calibration*: H-L | 1 | M: 12/12  R: 28/38 | multiple cross-validation |
| **Berlin score**:  Maier | 1997  (1978-91) | Germany | VLGA/BW  <1500g and <33w | 572  (31%^†^) | NICU admission | mortality- discharge | classification | *discrimination*: AUC  *calibration*: H-L  *accuracy*: at 21 points; % correct classification, sens, spec, npv, ppv | 1 | M: 11/12  R: 33/38 | split sample  subsequent validation: 1 study[[2]](#footnote-3) |
| Sulkes | 1998  (1980-90) | Israel | VLBW | 423  (43.7%) | stillbirths included | mortality- 1 year | other | *calibration*: H-L, Pearson (deviance)  *accuracy*: sens, spec, % correct survivors , nonsurvivors  *range* | 3 | M: 10/12  R: 32/38 | none |
| Fowlie | 1998  (1988-90) | UK | VLGA/BW  <1500g or <31w | 297  (14.5%) | 3d | mortality- discharge | clinical decisions | *discrimination*: AUC  *calibration*: H-L  *accuracy*: ppv at various levels | 9 | M: 9/12  R: 34/38 | none |
| Zernikow | 1998  (1990-96) | Germany | VLGA/BW  <1500g or <32w | 890  (8.3%) |  | mortality- 28d | other | *discrimination*: AUC  *range* | 2 | M: 10/12  R: 28/38 | temporally separate sample |
| **Draper grid**:  Draper | 1999  (1994-97) | UK | VLGA  22-32w | 3760  (19.6%) | stillbirths included | mortality- discharge | clinical decisions | *calibration*: H-L  *range* | 5 | M: 10/12  R: 27/38 | subsequent validation: 1 study |
| **SNAP-II** and **SNAPPE-II**:  Richardson | 2001  (1995-98) | CAN, USA | VLBW^*^  <=1500g | 25429  (3.9%^*)^ | NICU admission | mortality- discharge | case-mix adjustment | *discrimination*: AUC  *calibration*: H-L  *range* | 2 | M: 12/12  R: 34/38 | validated in split sample and geographically separate regions  subsequent validation: 4 studies |
| **NEOMOD**:  Janota | 2001  (1995-98) | Czech Republic | VLGA/BW  <1500g and <31w | 142  (16.9%) | NICU admission | mortality- 28d and discharge | case-mix adjustment  clinical decisions | *discrimination*: AUC  *(calibration*: H-L  *accuracy*: sens, spec) | 2 | -- | subsequent validation: 1 study[[3]](#footnote-4) |
| Gera | 2001  (1998) | India | VLBW  500-1500g | 115  (40.9%) | live birth | “early” and “late” neonatal mortality | other | *accuracy*: sens, spec | 2 | M: 7/12  R: 23/38 | none |
| **CRIB-II**:  Parry | 2003  (1998-99) | UK | VLGA  <=32w | 3027  (7.9%) | NICU admission | mortality- discharge | case-mix adjustment | *discrimination*: AUC, Cox  *calibration*: H-L | 1 | M: 11/12  R: 33/38 | split-sample (different centers)  subsequent validation: 6 studies[[4]](#footnote-5) |
| **NECOSUR**:  Marshall | 2005  (2000-03) | South America | VLBW  500-1500g | 1801  (26.6%) | before NICU admission | mortality- discharge | case-mix adjustment | *discrimination*: AUC  *calibration*: H-L | 1 | M: 12/12  R: 30/38 | split sample and cross-validation |
| Evans | 2007  (1998-2001) | AUS, NZ | VLGA/BW  <1500g or <32w | 11215  (6.8%) | NICU admission | mortality- discharge | case-mix adjustment | *discrimination*: AUC  *calibration*: H-L | 1 | M: 12/12  R: 31/38 | temporally separate sample |
| Ambalavanan | 2008  (2000-03) | USA | VLBW  401-1500g, resp. failure | 420  (52%) | 4h | mortality or BPD - discharge | classification | *discrimination*: c statistic  *accuracy*: at 95% sens; PPV, NPV, spec | 2 | M: 10/12  R: 27/38 | none |
| Basu | 2008  3 years | India | VLBW  <1500g | 260  (33.1%) | 12h | mortality- discharge | clinical decisions | *accuracy*: % predicted | 1 | M: 10/12  R: 22/38 | none |
| Almeida | 2008  (2004-05) | Brazil | VLGA/BW  400-1500g and 23-33w | 579  (15.9%) | 72h | mortality- 168h | case-mix adjustment | *calibration*: H-L | 1 | M: 10/12  R: 21/38 | none |
| **SAWS** and **SAW**:  Rosenberg | 2008  (1998-2003) | Bangladesh  Egypt | VLGA  <33w | 467  (50.5%) | 48h | mortality- discharge | case-mix adjustment  clinical decisions | *discrimination*: AUC, Cox  *calibration*: H-L  *range* | 2 | M: 11/12  R: 38/38 | bootstrap and validated in small, geographically separate sample (Nepal) |
| **PREM score**:  Cole | 2010  (2000-04) | UK | VLGA  22-31w | 4838  (18.3%) | stillbirths included | survival - term age | clinical decisions | *discrimination*: AUC  *calibration*: H-L  *range* | 3 | M: 11/12  R: 35/38 | 1 model validated in geographically separate region, 1 in mixed development / separate sample |
| Ballot | 2010  (2006-07) | South Africa | VLBW  <1501g | 448  (25.4%) | NICU admission | mortality- discharge | case-mix adjustment | *accuracy*: % predicted | 1 | M: 10/12  R: 27/38 | none |
| ELGA/BW (born at <28 weeks gestational age and/or <1000g birth weight) | | | | | | | | | | | |
| Herschel | 1982  (1977-80) | USA | ELGA  24-28w | 135  (50.3%) | live birth | survival – discharge | other | *accuracy*: % correct classification | 1 | M: 10/12  R: 11/38 | none |
| Zarfin | 1986  (1980-81) | CAN | ELBW  <801g | 106  (51.7%) | 8h | mortality- 24h to 28d | other | *accuracy*: at 0.50; % correct all, survivors, nonsurvivors  *range* | 3 | M: 10/12  R: 22/38 | none |
| Amon | 1987  (1981-85) | USA | ELBW  <1000g | 476  (59.6%) | live birth | survival - discharge | other | *accuracy*: % correct survivors, nonsurvivors | 1 | M: 9/12  R: 20/38 | none |
| Tyson | 1996  (1994-95) | USA | ELBW  501-800g | 947  (33.5%) | starting mechanical ventilation | mortality- discharge | case-mix adjustment  clinical decisions | *discrimination*: AUC  *accuracy*: at 0.50; sens, spec, % correct all, survivors, nonsurvivors  *range* | 1 | M: 12/12  R: 32/38 | split-sample |
| Doyle | 2001  (1991-92) | AUS | ELGA  23-27w | 401  (43.9%) | live birth | mortality- 5y | clinical decisions | *calibration*: H-L | 2 | M: 10/12  R: 22/38 | none |
| Ambalavanan | 2001  (1990-96) | USA | ELBW  <1000g | 810  (34%) | NICU admission | mortality | case-mix adjustment | *discrimination*: AUC  *discrimination/*  *calibration/accuracy*: r^2^  *accuracy*: at 80% sens; spec, PPV, NPV | 4 | M: 11/12  R: 28/38 | split-sample |
| Locatelli | 2005  (1998-2002) | Italy | ELGA/BW  <750g and <34w | 59  (49.2%) | live birth | survival - discharge | other | *discrimination / calibration/accuracy:* R^2^  *accuracy*: sens, spec, ppv, npv | 1 | M: 9/12  R: 28/38 | none |
| Ambalavanan | 2005  (1998-2003) | USA | ELBW  401-1000g | 8608  (35%) | live birth | mortality | clinical decisions | *discrimination*: AUC  *calibration*: H-L  *accuracy*: at 0.50 and 0.90 sens; PPV, NPV, spec | 10 | M: 12/12  R: 31/38 | split-sample |
| Ambalavanan | 2006  (1996-98) | Canada, Australia, NZ, US, Hong Kong | ELBW  500-999g | 1036  (41.9%) | 8d | mortality or NDI – 18m | other | *accuracy*: sens, spec, PPV, NPV, accuracy  *range* | 6 | M: 12/12  R: 37/38 | split-sample and cross-validation |
| Forsblad | 2007  (1995-2001) | Sweden | ELGA  25w | 92  (21%) | live birth | survival - 180d | other | *calibration*: H-L  *range* | 4 | M: 9/12  R: 30/38 | none |
| **NICHD**:  Tyson | 2008  (1998-2003) | USA | ELGA/BW  401-1000g and 22-25w | 3702  (42%) | starting mechanical ventilation | mortality- 18m | clinical decisions | *discrimination*: AUC | 2 | M: 12/12  R: 34/38 | bootstrap |
| Forsblad | 2008  (2000-02) | Sweden | ELGA  23 and 24w | 156  (53.8%) | live birth | survival - 180d | other | *calibration*: H-L  *range* | 3 | M: 9/12  R: 30/38 | none |
| Gargus | 2009  (1998-2001) | USA | ELBW  401-1000g | 5250  (34.4%) | live birth | mortality- 18m | other | *discrimination/*  *calibration/accuracy*: R^2^ | 3 | M: 10/12  R: 27/38 | none |

The 41 development studies were heterogeneous in population, aim, performance measures, validation, and quality. There were 28 studies for a general VLGA/BW population and 13 studies specific to ELGA/BW infants, dating from 1982-2010. The number of infants ranged from 59 to 12960 and the mortality rate from 6.8% to 59.6%. Further details of these studies are given in Table S3.

1. 2 studies include authors of the original CRIB score, 1 other mentions involvement with the CRIB study [↑](#footnote-ref-2)
2. same authors as original study, added a second center and temporally separate [↑](#footnote-ref-3)
3. same authors, geographically separate population [↑](#footnote-ref-4)
4. one study mentions affiliation with the CRIB-II study [↑](#footnote-ref-5)
