# Supplementary material for: Prediction of Mortality in Very Premature Infants: A Systematic Review of Prediction Models
Source: PLoS One. 2011 Sep 8;6(9):e23441. doi: 10.1371/journal.pone.0023441 (PMC3169543; doi:10.1371/journal.pone.0023441)
Supplement: Table S3 — Detailed summary of the 41 included development studies. (DOC) [file pone.0023441.s003.doc]

**Table S3**: **Detailed summary of the 41 included development studies**

| VLGA/BW | | | | | | |
| --- | --- | --- | --- | --- | --- | --- |
| Study | setting, surfactant, and antenatal steroids | population | outcome, time of prediction, purpose | model and validation | performance | conclusion |
| The relationship of Apgar scores, gestational age, and birthweight to survival of low-birthweight infants.  Behnke M, Carter RL, Hardt NS, Eyler FD, Cruz AC, Resnick MB.  Am J Perinatol. 1987 Apr;4(2):121-4. | University of Florida College of Medicine, USA  June 1974 to July 1980  **surfactant**: not reported  **antenatal steroids**: not reported | 500-1800g  n = 748 (161 deaths)  **age at inclusion**: live birth  **resuscitation policy**: not reported  **inclusion criteria**: live born, inborn  mean birth weight:  survivors = 1429g (SD 273)  nonsurvivors = 977g (SD 337)  mean gestational age:  survivors = 31.7w (SD 2.5)  nonsurviors = 27.2w (SD 3.0) | **outcome**: survival to 28 days  **mortality**: 21.5%  **time of prediction**: at birth  **purpose**: Our purpose was to assess the relationship between 1 and 5 minute Apgar scores and survival, and to evaluate all combinations of the four variables as outcome predictors. | **model**: logistic regression  linearity: not reported  *final model not reported*  *Final variables:*  GA  1 min Apgar  5 min Apgar  **validation**: not done | cutoff: 0.50  1 min Apgar:  correct prediction = 80%  Sn = 92%  Sp = 38%  false pos = 16%  false neg = 45%  5 min Apgar:  correct prediction = 84%  Sn = 94%  Sp = 47%  false pos = 14%  false neg = 33%  birthweight:  correct prediction = 85%  Sn = 94%  Sp = 51%  false pos = 13%  false neg = 30%  GA:  correct prediction = 86%  Sn = 94%  Sp = 55%  false pos = 12%  false neg = 27%  BW, GA, A1, A5:  correct prediction = 86%  Sn = 94%  Sp = 57%  false pos = 11%  false neg = 27%  GA, A1, A5:  correct prediction = 87%  Sn = 95%  Sp = 58%  false pos = 11%  false neg = 24%  **range**: not reported | “Our conclusion is that Apgar scores do not reflect the same clinical states in term and premature infants. For example, when muscle tone is evaluated, premature infants may have a lower score in this category because of their physiologic immaturity, not necessarily because of pathology as a low tone score suggests in term infants. Until the etiology of the relationships between scores and outcome can be clarified further, we do not recommend that Apgar scores be applied to low birthweight infants with the same implications for prognosis as for term babies. However, in combination with gestational age, they can provide a predictive model for mortality superior to that available with any single variable.” |
| Prediction of outcome shortly after delivery for the very low birthweight (less than or equal to 1500 g) infant.  Patterson CC, Halliday HL.  Paediatr Perinat Epidemiol. 1988 Jul;2(3):221-8. | Royal Maternity Hospital, Belfast, UK  January 1978-December 1985  **surfactant**: not reported  **antenatal steroids**: not reported | <= 1500g  n = 387 (86 deaths)  development n = 287  validation n = 100  **age at inclusion**: admission to NICU (stillbirths and delivery-room deaths excluded)  **resuscitation policy**: not reported  **inclusion criteria**: inborn only (including intrauterine transfers); lethal congenital malformations and delivery room deaths excluded  mean birthweight:  survivors: 1259g (SD 192)  deaths: 1054g (SD 251)  mean gestational age:  survivors: 30.2w (SD 2.4)  deaths: 27.4w (SD 1.9) | **outcome**: hosptial mortality  **mortality**: 22.2%  **time of prediction**: admission to NICU  **purpose**: The probabilities derived from the model are tabulated and may help paediatricians in maternity units without neonatal intensive care facilities to decide which very low birthweight infants to transfer for additional care. | **model**: logistic regression  linearity: not reported  *final model not reported*  *Final variables:*  gestational age  Apgar at 5 minutes  signs of respiratory distress within 1 hour of birth  **validation**: split sample | cutoff: 0.50  [in the validation group] the model correctly identified 89% of survivors (68 out of 76) but only 54% of deaths (13 out of 24)  [in the validation group] 47% of survivors and no non-survivors had a predicted risk of 1/20 [however, in the development group, 4/86 nonsurvivors had this predicted risk]  **range**: <5%-90% | *No specific conclusion section, last paragraph of article:*  “Previous authors have noted physicians' lack of appreciation of their own nursery's survival rates. We hope that, in addition to helping determine appropriate care for the very low birthweight infant, studies such as ours may enable physicians looking after such infants to provide parents with reliable quantitative estimates of prognosis.” |
| Variability in 28-day outcomes for very low birth weight infants: an analysis of 11 neonatal intensive care units  Horbar JD, McAuliffe TL, Adler SM, Albersheim S, Cassady G, Edwards W, Jones R, Kattwinkel J, Kraybill EN, Krishnan V, et al.  Pediatrics. 1988 Oct;82(4):554-9. | 11 NICUs in the US, Canada, and England: neonatologists who expressed interest in participating and voluntarily submitted data  1983-1984  **surfactant**: not reported  **antenatal steroids**: “obstetrical practices may vary widely from center to center, particularly with respect to the use of prenatal steroids”  **race**: 75.5% white, 18.2% black, 6.3% other (s) | 701-1500g  n = 1776 (264 deaths, 720 dead or in need of supplemental oxygen)  **age at inclusion**: live birth  **resuscitation policy**: not reported (multicenter)  **inclusion criteria**: all live births  *n given in each birth weight category* | **outcome**: survival, and survival without the need for supplemental oxygen, by day 28  **mortality**: 14.8%  **time of prediction**: not specified, all variables are available at birth  **purpose**: “Because comparisons are confounded by population differences, we determined the effects of birth weight, gender, and race on outcome and adjusted for these factors in the analysis.” | **model**: linear regression  linearity: log-linear models  *final model not reported*  *Final variables:*  birth weight  gender  race  center  **validation**: not done | model survival:  H-L p=0.13  model survival without supplemental oxygen:  H-L p=0.17  **range**: *not reported; gives predicted outcomes per center* | “In conclusion, the rates of survival and survival without supplemental oxygen on day 28 after birth for inborn infants weighing 701 to 1500g vary significantly among neonatal intensive care units. Both outcomes are influenced by birth weight, gender, and race. The sources of the residual variability are currently uncertain. They may include differences in the distribution of gestational ages or disease severity among centers or could be related to differences in obstetrical and neonatal care. Prospective studies are needed to identify the causes for variation in outcome of very low birth weight infants. These studies should include prognostic stratification of the patients based on physiologic status and disease severity.” |
| Development and validation of a multivariate predictor of mortality in very low birth weight.  Ales KL, Frayer W, Hawks G, Auld PM, Druzin ML.  J Clin Epidemiol. 1988;41(11):1095-103. | a tertiary care hospital in NY – Cornell  USA  development 1 Jan 1984 - 31 Dec 1985; validation 1 Jan 1986 – 31 Dec 1986  **surfactant**: not reported  **antenatal steroids**: tested as a variable, number treated not reported  **race**: 48% black, 35% white, 17% other (NS) | <=1500g  n = 158 (30% mortality)  **age at inclusion**: live births (excluded dead before maternal admission and intrapartum deaths)  **resuscitation policy**: not reported  **inclusion criteria**: singleton, inborn;  excluded fatal anomalies  *birth weight/GA distributions given in a table* | **outcome**: death during the first hospitalization  **mortality**: 30%  **time of prediction**: at birth  **purpose**: prognostic model for viable liveborn singletons, risk stratification for randomized controlled trials | **model**: “multivariate techniques”  linearity: not reported  probability = {1 + e-(6.6019 - .4401 (5 min Apgar) - .0042(BW))}-1  **validation**: temporally separate validation set | cutoff not reported  “the model with the maximal predictive accuracy (84.5%) utilized birth weight and 5-min Apgar... the model using crown-heel length and Apgar score at 5 minutes had a similar level of predictive accuracy (83.7%)”  development:  Wilcoxon statistic = 0.85 (SE 0.04)  point of inflection = 0.75  at point of inflection, likelihood ratio = 44.9 (likelihood of having probability > 0.75 in those who actually died)  validation:  Wilcoxon statistic = 0.86 (SE 0.07)  model χ^2^ = 73.02, df=2, R = 0.596  **range**: (graph) approx. 1% to 97% | “In conclusion, this model underscores the importance of stratification before randomization according to the calculated probability of mortality, rather than according to birth weight alone. Given the ability of the model to generate reasonable estimates of mortality, the information might be used in the clinical setting to assist parents and physicians in individualized decision-making processes for a given infant. The ability to predict long term morbidity was beyond the scope of this study, but should be pursued in future research.” |
| Predicting death from initial disease severity in very low birth weight infants: a method for comparing the performance of neonatal units.  Tarnow-Mordi W, Ogston S, Wilkinson AR, Reid E, Gregory J, Saeed M, Wilkie R.  BMJ. 1990 Jun 23;300(6740):1611-4. | two United Kingdom hospitals  1 January 1986 to 31 May 1989 (hospital A)  1 November 1983 to 14 September 1986 (hospital B)  **surfactant**: not reported  **antenatal steroids**: not reported | < 1500 g and received artificial ventilation via an endotracheal tube at some time in the first 72 hours of life  n = 262  hospital A: 130 (23 deaths)  hospital B: 132 (32 deaths)  **age at inclusion**: 12h  **resuscitation policy**: not reported  **inclusion criteria**: Infants who died within the first 12 hours of life or had severe congenital malformations were excluded.  mean birthweight:  hospital A = 1143g (SD 220)  hospital B = 1044g (SD 260)  mean gestational age:  hospital A = 29.2w (SD 2.6)  hospital B = 28.3w (SD 2.3) | **outcome**: death in hospital  **mortality**: 21.0%  **time of prediction**: 12h  **purpose**: comparing mortality between two neonatal units | **model**: logistic regression  linearity: not reported  log (R/l-R)=54.1 (20.4) x 1 - 0.11 (0.11) x gestation  - 0.36 (0.41) x sex  - 0.004 (0.001) x birth weight  - 0.36 (0.47) x respiratory distress syndrome  + 0.05 (0.01) x FiO2 (+ 2.71 (1.4) if FiO2 missing)  - 6.67 (2.68) x pH (-47.2 (19.5) if pH missing)  - 0.23 (0.21) x year of birth  - 1.89 (0.79) x hospital  **validation**: multiple models created based on 4 traditional risk factors then adding the respiratory variables stepwise. Model was tested at each step on hospital B  The data was then pooled and new regression models derived. | Traditional risk factors (sex, birth weight, gestation, respiratory distress syndrome) :  Sn = 31% (10/32)  Sp = 94% (94/100)  Traditional risk factors + mean a/AO2 ratio:  Sn = 59% (19/32)  Sp = 87% (87/100)  Traditional risk factors + mean a/AO2 ratio + mean pH:  Sn = 75% (24/32)  Sp = 76% (76/100)  FINAL MODEL:  cutoff: 0.50  Traditional risk factors + mean FiO2 + mean pH:  Sn = 81% (26/32)  Sp = 74% (74/100) PPV = 50% (26/52)  NPV = 93% (74/80)  % correctly classified: 100/132 = 76%  **range**: *not reported; gives predicted number of deaths per hospital.* | “Crude comparisons of hospital mortality can be highly misleading. Reliable assessment of neonatal outcome is impossible without correcting for major risk factors, particularly initial disease severity. International agreement on a minimum core dataset of clinical and physiological information could improve neonatal audit and help to identify effective treatments and policies.” |
| Predicting mortality risk for infants weighing 501 to 1500 grams at birth: a National Institutes of Health Neonatal Research Network report.  Horbar JD, Onstad L, Wright E.  Crit Care Med. 1993 Jan;21(1):12-8. | seven centers participating in the NICHHD Neonatal Research Network during the first 2 years of routine data collection  USA  November 1, 1987 to October 31, 1989  **surfactant**: “A major change in neonatal practice has occurred since our data were collected, namely, the introduction of surfactant therapy into routine care.”  **antenatal steroids**: 5.2% of deaths and 9.6% of survivors, 8.5% overall  **race**: black 61.4% (s) | 501 – 1500g  n = 3646 (24.7% mortality)  n development = 1823  n validation = 1780  + 43 missing data  **age at inclusion**: not reported  **resuscitation policy**: not reported  **inclusion criteria**: *no further inclusion/exclusion given*  mean birth weight = 1060g (SD 282)  *summary statistics for gestational age not reported* | **outcome**: Final disposition was tracked until the infants were discharged either to home or to a chronic care facility.  **mortality**: 24.7%  **time of prediction**: admission to NICU  **purpose**: “To develop and evaluate a model that predicts mortality risk based on admission data for infants weighing 501 to 1500g at birth, and to use the model to identify neonatal ICUs where the observed mortality rate differs significantly from the predicted rate.” | **model**: logistic regression  linearity: not reported  birth weight -0.422  size for GA -0.491  race -0.450  male gender 0.656  1 min Apgar 0.971  constant 2.606  **validation**: randomized sample | cutoff of 0.50 :  Sn = 0.50  false pos = 0.09  cutoff of 0.25 (optimal)  Sn = 0.76  false pos = 0.24  dev:  Sn = 0.49  Sp = 0.93  PPV = 0.69  NPV = 0.85  correct classification = 0.82  AUC = 0.82 (SE 0.01)  goodness of fit χ^2^=14.3  p=0.07  val:  Sn = 0.50  Sp = 0.92  PPV = 0.68  NPV = 0.85  correct classification = 0.82  AUC = 0.82 (SE 0.01)  goodness of fit χ^2^=15.4  p=0.06  birthweight only:  Sn 7% lower  H-L χ^2^=22.0, p=0.05  “other performance measures were similar”  **range**: <10% - <90% | “Mortality risk for infants weighing 501 to 1500g can be predicted based on admission factors. However, until more accurate predictive models are developed and validated and the relationships between care practices and outcomes are better understood, such models should not be relied on for evaluating the quality of care provided in different neonatal ICUs.” |
| The CRIB (clinical risk index for babies) score - a tool for assessing initial neonatal risk and comparing performance of neonatal intensive-care units.  The International Neonatal Network.  Lancet. 1993 Jul 24;342(8865):193-8. | four UK tertiary hospitals  July 1, 1988 - June 30, 1990  **surfactant**: “The cohort was recruited before the widespread introduction of surfactant therapy.”  **antenatal steroids**: not reported | birthweight <= 1500 g *or* gestational age <31w  n = 1198  n development =735 (187 deaths)  n validation =463 (104 deaths)  **age at inclusion**: NICU admission  **resuscitation policy**: not reported  **inclusion criteria**: all infants without inevitably lethal congenital malformations admitted to four UK teaching hospital neonatal units  *n in each birth weight category given in table, summary statistics for gestational age not reported* | **outcome**: hospital mortality  **mortality**: 24.3%  **time of prediction**: 12h  **purpose**: “...the CRIB score could be used as a surrogate measure of outcome for randomised trials of obstetric interventions. It could also assist individual clinical audit... However, we would strongly discourage its use as a justification for withdrawing intensive care in an individual severely ill infant. CRIB is primarily a tool for comparing populations and cannot provide individual prognoses accurately enough to dictate individual decisions.” | **model**: logistic regression  linearity: not reported  *presented as a score*:  birth weight (g):   | >1350 | 0 | | --- | --- | | 851-1350 | 1 | | 701-850 | 4 | | <=700 | 7 |   gestation (wk)   | >24 | 0 | | --- | --- | | <24 | 1 |   congenital malformations   | none | 0 | | --- | --- | | not acutely life threatening | 1 | | acutely life threatening | 3 |   maximum base excess in first 12h (mmol/L)   | > -7.0 | 0 | | --- | --- | | -7.0 to -9.9 | 1 | | -10.0 to 14.9 | 2 | | <= -15.0 | 3 |   minimum appropriate FiO_2_ in first 12h   | <= 0.40 | 0 | | --- | --- | | 0.41-0.60 | 2 | | 0.61-0.90 | 3 | | 0.91-1.00 | 4 |   maximum appropriate FiO_2_ in first 12h   | <= 0.40 | 0 | | --- | --- | | 0.41-0.60 | 1 | | 0.61-0.90 | 3 | | 0.91-1.00 | 5 |   **validation**: separate sample from 4 different hospitals, similar geographic area | CRIB  dev  H-L χ^2^=16.84, df=1, p=0.078  AUC = 0.92 (SE 0.04)  val  AUC = 0.90 (SE 0.05)  at Sp = 95%  Sn = 51%  val subgroup BW <1500g  AUC = 0.90  birthweight  development:  AUC = 0.79 (SE 0.02)  difference between CRIB and birthweight: p=0.001  validation:  AUC = 0.78 (SE 0.03)  difference between CRIB and birthweight: p=0.03  validation subgroups: BW<1500g continuous  AUC = 0.79  250g intervals  AUC = 0.75  **range**: *not reported; mortality by category of score is given graphically* | *No specific conclusion given. Last paragraph of abstract :*  “CRIB is a robust index of initial neonatal risk that is more accurate than birthweight and simple enough for routine use. International comparisons of staffing policy and organisation of neonatal intensive care units are needed to investigate the reasons for variations in performance.” |
| An index for early assessment of neonatal survival in low birthweight infants.  Carter RL, Behnke M, Ariet M, Dickman H, Resnick MB.  Am J Perinatol. 1995 Nov;12(6):392-5. | Shands Teaching Hospital, U of Florida  USA  July 1, 1980 - March 3, 1983  **surfactant**: “Surfactant has markedly reduced mortality rates among infants with true RDS, but it is not 100% effective even in the weight groups in which it is considered successful. So, although the use of surfactant may have altered the effect of this diagnosis somewhat, it would not have eliminated the effect totally.”  **antenatal steroids**: not reported  **race**: white 56% (NS) | 500-1800g  n = 309 (76 deaths)  **age at inclusion**: not reported  **resuscitation policy**: not reported  **inclusion criteria**: receiving neonatal intensive care.  excluded 132 due to missing data  mean birthweight:  survivors = 1381g (SD 303)  nonsurvivors = 1012g (SD 337)  mean gestational age:  survivors = 31.3w (SD 2.7)  nonsurvivors = 28.0w (SD 2.7) | **outcome**: death before discharge  **mortality**: 24.6%  **time of prediction**: 24 hours  **purpose**: “The purpose of this study was to develop a survival index applicable to premature infants” | **model**: logistic regression  linearity: not reported  birthweight -0.0024  family density -0.3077  respiratory distress syndrome 1.9809  prenatal ultrasound examination -1.3486  spontaneous premature labor 1.3330  appropriate birthweight for gestational age -1.2490  convulsions 3.4460  gestational age -0.2617  1 minute Apgar -0.1807  maternal age –  intercept: 10.9750  **validation**: not done | cutoff: 0.50  of the 233 survivors, 221 were predicted to live  of the 76 who died, 52 were predicted to die  Sn = 94.8%  Sp = 68.4%  88.3% correct classification  of the 245 predicted to live, 221 lived  PPV = 90.2%  of the 64 predicted to die, 52 died  NPV = 81.3%  **range**: *not reported* | “We offer the NSI as an index of neonate condition on admission to the NICU. It could also be used as a predictor of mortality but first must be validated as such in time- and location-specific validation studies. In either case, the NSI is intended to supplement other information used in making clinical judgments. It is hoped that the model developed herein produces useful additional input into the clinician's usual processing of information.” |
| Changes in survival patterns of very low-birth-weight infants from 1980 to 1993.  Roth J, Resnick MB, Ariet M, Carter RL, Eitzman DV, Curran JS, Cupoli JM, Mahan CS, Bucciarelli RL.  Arch Pediatr Adolesc Med. 1995 Dec;149(12):1311-7. | Regional Perinatal Intensive Care Centers, Florida, USA  1980-1993  **surfactant**: not reported  **antenatal steroids**: not reported  **race**: black 55.9%, white 44.1% (s) | VLBW (500-1500g)  n = 12960 (80.8% survival)  *n for each model is not reported*  **age at inclusion**: admission to perinatal intensive care (excluding stillbirths and delivery room deaths)  **resuscitation policy**: not reported  **inclusion criteria**: singleton;  excluded delivery room deaths  excluded infants who were not transferred to an RPICC  *distribution of birth weights in sample is given in table* | **outcome**: mortality - hospital  **mortality**: 19.2%  **time of prediction**: not specified, data available at birth  **purpose**: “To determine changes in survival patterns among VLBW infants between 1980-1993” | **model**: log linear regression model  linearity: squared coefficients tested  *coefficients given for each of the 8 models*  *Final variables:*  birthweight  year  year^2^  birthweight x year  birthweight x year^2^  **validation**: not done | B = black, W = white  M = male, F = female  I = inborn, T = transfer  scaled deviance:  B/M/I = 196.9  B/M/T = 200.7  B/F/I = 238.6  B/F/T = 163.2  W/M/I = 212.8  W/M/T = 217.0  W/F/I = 217.5  W/F/T = 214.5  **range**: (graphs) ranges from approx. 20%-80% to 85-100% in most recent years. | “These results illustrate the value of taking into account race, sex, and transport status in efforts to understand the contribution that neonatal intensive care of extremely low birth weight infants makes toe the lowering of infant mortality, and of using mulitvariable statistical procedures to generate predicted survival probabilities for different subpopulations. These probabilities can be applied to (1) predicting survival for specific subgroups of extremely low birth weight infants, and (2) helping physicians develop clinical guidelines for extending care to infants at the threshold of viability.” |
| Factors associated with poor prognosis in very-low-birth-weight infants.  Ballot DE, Mohanlal P, Davies VA, Cooper PA.  S Afr Med J. 1996 Nov;86(11 Suppl):1457-60. | Johannesburg Hospital, South Africa  1 September 1992 - 1 September 1994  secondary analysis of infants enrolled in an international study using the CRIB score  **surfactant**: Surfactant was administered to 78 of 159 ventilated babies (49%)  **antenatal steroids**: “Antenatal steroids, including incomplete courses, had been administered to 17% of mothers in preterm labour.” | birth weight below 1501 g *and/or* gestational age less than 31 weeks  n = 231 (84% survived)  **age at inclusion**: not specified; probably 12h as in CRIB score  **resuscitation policy**: infants <1000g not routinely ventilated.  **inclusion criteria**: Infants weighing <1000g were excluded because they are not routinely ventilated.  Infants who were moribund on admission and treated conservatively were excluded.  mean birth weight = 1286g (SD 152)  mean gestational age = 30.9w (SD 2.2w) | **outcome**: death, death or impairment at 28d  **mortality**: 16%    **time of prediction**: not specified, uses max PaCO2 at 72h  **purpose**: To evaluate predictors of poor outcome, including the CRIB score, in a local population of very-Iow-birth-weight (VLBW infants, in order to provide guidelines for selection of these babies for expensive tertiary care. | **model**: logistic regression  linearity: not reported  death:  logit (P) = 5.149 + 0.0292 max PaCO2 + 0.0354 FiO_2_  **validation**: not done | death:  goodness of fit χ^2^ p=0.98  **range**: *not reported* | “One method of selection of infants for expensive tertiary care is on the basis of predicted outcome. Birth weight remains a reasonable basis for this selection, but the inclusion of other factors, such as oxygen requirement, would improve accuracy. The CRIB score was not a suitable means to select infants in the local context, but may be of value in international comparisons.” |
| Hospital and patient characteristics associated with variation in 28-day mortality rates for very low birth weight infants. Vermont Oxford Network.  Horbar JD, Badger GJ, Lewit EM, Rogowski J, Shiono PH.  Pediatrics. 1997 Feb;99(2):149-56. | 62/68 Vermont Oxford Network NICUs (voluntary participation of NICUs in North America, Europe, Asia, and Australia)  1991-2  **surfactant**: 53% percent of the infants received surfactant treatment (interquartile range 43% to 62%).  **antenatal steroids**: “The frequency of antenatal steroid use varied among the participating institutions” (interquartile range 10% to 31%)  **ethnicity**: black 28.9% | 501-1500g  n = 7672 (14.7% mortality)  **age at inclusion**: birth  **resuscitation policy**: not reported  **inclusion criteria**: including all inborn infants, including those who died before NICU admission, and all transfers before day 28 of life  transfers were followed until discharge  excluded institutions which were missing 10% or more of data  mean birth weight = 1059g (SD 281)  mean gestational age = 28.5w (SD 2.9) | **outcome**: death on or before the 28^th^ day of life  **mortality**: 14.7%  **time of prediction**: birth (before NICU admission)  **purpose**: “The purpose of the present study is to identify patient and hospital characteristics responsible for differences among NICUs in mortality rates for very low birth weight infants.” | **model**: logistic regression  linearity: not reported  *final model not reported*  *Final variables:*  birth weight  1-minute Apgar  race  gender  mode of delivery multiple birth  antenatal steroid use  small size for gestation age  prenatal care  major birth defect  **validation**: multiple cross-validation using 10 equal subsamples. | H-L goodness-of-fit = 10.5, df=8, p=0.23  AUC = 0.87 (SE 0.009)  mean R^2^ = .30  **range**: *not reported* | “In summary, we found differences among NICUs in neonatal mortality rates for very low birth weight infants that were not explained by differences in the characteristics of the infants treated. This suggests that the effectiveness of care varies among units. A major goal of the Vermont Oxford Network is to help participating NICUs improve the effectiveness of their care through a coordinated program of randomized trials, outcomes research, and quality improvement projects. In addition, we found that neither the annual volume of very low birth weight infants treated at a NICU, nor the presence of a pediatric residency training program were associated with differences in mortality risk. This finding is of importance because the current trend toward deregionalization of neonatal intensive care will result in a larger proportion of infants receiving their treatment at smaller, nonteaching units. We found no evidence to suggest that this trend will result in higher mortality rates for very low birth weight infants.” |
| Comparison of mortality risk: a score for very low birthweight infants.  Maier RF, Rey M, Metze BC, Obladen M.  Arch Dis Child Fetal Neonatal Ed. 1997 May;76(3):F146-50; discussion F150-1. | Virchow-Klinikum,  Humboldt-Universität  Berlin, Germany  1978 to 1991  **surfactant / antenatal steroids**: “In none of the infants had surfactant been administered before admission... The rationale for defining these two periods were major changes in perinatal and neonatal conditions, such as.... surfactant therapy for respiratory distress syndrome... Variations in use of antenatal steroids may lead to substantial variations in gestation specific severity of illness.” | VLBW (below 1500g) and < 33w  n = 572  n development = 396  n validation = 176 (31% mortality)  **age at inclusion**: admission to NICU  **resuscitation policy**: not reported  **inclusion criteria**: included congenital malformations, outborn  excluded 112 infants due to missing data or gestational age >33w  median birth weight = 1150g (5^th^% 680; 95^th^% 1470)  median gestational age = 29.3w (5^th^% 25.0; 95^th^% 35.0) | **outcome**: death in hospital  **mortality**: 31% (validation)  **time of prediction**: at NICU admission  **purpose**: “To develop and evaluate a score which quantifies mortality risk in very low birthweight (VLBW) infants (birthweight below 1500 g) at admission to the neonatal intensive care unit” | **model**: logistic regression  linearity: not reported  birthweight  respiratory distress syndrome  Apgar score at 5 minutes  artificial ventilation  base excess at admission  constant  Equation to predict mortality:  Pr(mortality) = 1 / (1 + exp (-(- 0.37- 0.84*BW + 0.71*RDS - 0.55*APGAR + 0.53*VENT + 0.37*BE)))  *also presented as a score*  **validation**: randomized sample | validation cohort  AUC = 0.86  cutoff = 21 points  Sn = 0.85  Sp = 0.73  NPV = 0.92  PPV = 0.55  correct classification = 76%  cutoff = 18 points  PPV = 0.44  NPV = 0.98  cutoff = 29 points  PPV = 0.80  NPV = 0.78  development:  H-L p=0.56  validation :  H-L p=0.99  recent:  H-L p=0.71  same model without variable “artificial ventilation”:  AUC = 0.81  **range**: *not reported, about 50% of infants charaterized as low risk, about 10% as high risk.* | “The present score is robust, easily obtainable at admission, and permits early randomisation based on mortality risk.” |
| Mortality in very low birth weight infants. The Beilinson Medical Center Experience, 1980-1990.  Sulkes J, Gabbay U, Hod M, Merlob P.  Is J Obst Gynecol 1998; 9(4): 248-256. | Beilinson Medical Center, Petah Tikva, Israel  1980-1990  **surfactant**: “during this period surfactant treatment had not yet been introduced”  **antenatal steroids**: “Major changes in neonatal practice have occurred since our data were collected, namely, the introduction of surfactant therapy into routine care, the use of corticoids...” | VLBW  n = 423 (185 deaths: 24 intrapartum, 128 early neonatal, 15 late neonatal, 18 infant deaths)  **age at inclusion**: stillbirths included (prior to delivery)  **resuscitation policy**: not reported  **inclusion criteria**: singleton;  excluded antepartum deaths;  excluded major congenital anomalies or chromosomal aberrations  mean birth weight = 1073g (SD 278)  mean gestational age = 29w (33.2)*?* | **outcome**: total mortality to 1 year of age  **mortality**: 43.7%  **time of prediction**: pre-delivery (1 model)  birth (1 model)  post-delivery (1 model)  **purpose**: “To evaluate the potential factors influencing mortality in a cohort of 423 singleton VLBW infants born at Beilinson Medical Center during the years 1980-90 (presurfactant era).” | **model**: logistic regression  linearity: not reported  pre-delivery:  intercept: 16.6345  appropriateness for gestational age 0.8768  gestational age (week) -0.5595  mother's age (yr) -0.0558  PROM -0.5971  PIH -0.8559  fetal distress 1.2807  birth and post-delivery:  intercept: 28.0287  gestational age (week) -0.1265  mother's age (yr) -0.0646  PIH -1.2266  fetal distress 1.2097  birth weight (g) -0.00293  Apgar at the fifth minute -0.5537  year of birth -0.2010  + seizures 0.7964 (for post-delivery)  **validation**: not done | pre-delivery:  Sn = 65.3%  Sp = 90.2%  H-L χ^2^=7.95, p=0.45  **range**: 2%-98%  Pearson or deviance statistic = 314.55 < 345  birth and post-delivery:  H-L χ^2^=8.03, p=0.40  Pearson 240<264, deviance 164<264  44% of observed deaths predicted  94% of surviving newborns correctly classified  **range**: 3% - 48% | “The methodology can be adopted by other institutions to evaluate the risk of death in VLBW infants” |
| Predicting outcome in very low birthweight infants using an objective measure of illness severity and cranial ultrasound scanning.  Fowlie PW, Tarnow-Mordi WO, Gould CR, Strang D.  Arch Dis Child Fetal Neonatal Ed. 1998 May;78(3):F175-8. | six Scottish hospitals  1988 – 1990  **surfactant / antenatal steroids**: “Neonatal practice has also changed dramatically over the past 10 years with the introduction of antenatal steroids and surfactant treatment” | <1500g *or* <31w  n = 297 (43 deaths between 3 days of life and discharge)  **age at inclusion**: 3d  **resuscitation policy**: not reported  **inclusion criteria**: inborn; survived until the end of day 3. (428 born, 46 died in the first 3 days, 85 excluded due to missing data)  median birth weight = 1170g (range 560-1500)  median gestational age = 29w (range 24-37) | **outcome**: death between day 3 and hospital discharge  **mortality**: 14.5%  **time of prediction**: 3 days after birth  **purpose**: “To investigate the feasibility of developing an objective tool for predicting death and severe disability using routinely available data, including an objective measure of illness severity, in very low birthweight babies...focus on babies who have survived the immediate postnatal period.” | **model**: “regression techniques”  linearity: *not reported*  BW: Log odds death -2.0044 + (0.0034 * birth weight)  GA: Log odds death -10.7097 + (0.4423 * gestation)  CRIB72 + USS: Log odds death 4.0334 - (1.6584 * USS) - (0.4004 * CRIB72)  USS = dichotomous ultrasound appearance (0-2 or 3-4)  **validation**: not done | BW:  AUC = 0.70 (SE 0.05)  H-L χ^2^=9.32, p=0.50  GA:  AUC = 0.74 (SE 0.08)  H-L χ^2^=9.27, p=0.51  CRIB72 + USS:  AUC = 0.89 (SE 0.05)  H-L χ^2^=5.57, p=0.85  CRIB72 > 4 plus IVH grade 3 or 4:  PPV = 64%  CRIB72 = 2-4 plus IVH grade 3 or 4:  PPV = 50%  CRIB72 = 1 plus IVH grade 3 or 4:  PPV = 14%  CRIB72 = 0 plus IVH grade 3 or 4: none  CRIB72 > 4 plus IVH grade 0, 1 or 2:  PPV = 35%  CRIB72 = 2-4 plus IVH grade 0, 1 or 2:  PPV = 6%  CRIB72 = 1 plus IVH grade 0, 1 or 2:  PPV = 3%  CRIB72 = 0 plus IVH grade 0, 1 or 2:  PPV = 0%  **range**: *not reported* | “...incorporating objective measures of illness severity may improve current prediction of death and disability in premature infants. Our results suggest that assessment of illness severity permits a more accurate estimate of prognosis than use of birthweight, gestation, or cranial ultrasound scans alone. However, no single piece of evidence can provide a definitive estimate of prognosis for individual babies. Any score is based on population characteristics that may not pertain to the individual baby, relates to outcome data that are by definition already 'out of date,' and, when derived from relatively small cohorts, will give estimates which lie within wide confidence limits. Clinicians’ clinical judgment is a powerful tool in its own right and scoring systems can only provide an additional insight into prognosis. They should never be used in isolation to make clinical decisions, particularly about withdrawal of active treatment, and we would encourage clinicians to continue to use an holistic approach.” |
| Artificial neural network for risk assessment in preterm neonates.  Zernikow B, Holtmannspoetter K, Michel E, Pielemeier W, Hornschuh F, Westermann A, Hennecke KH.  Arch Dis Child Fetal Neonatal Ed. 1998 Sep;79(2):F129-34. | Vestische Kinderklinik Witten/Herdecke University, Germany  4 April 1990 - 24 October 1996  (training set 04-04-90 – 05-11-93  validation set  06-11-93 – 24-10-96)  **surfactant**: “It should be mentioned that in contrast to published reports, surfactant treatment... had already been introduced into routine care during the study period.”  **antenatal steroids**: not reported | gestational age <32 completed weeks *or* birthweight <1500g  n = 890  n training = 435 (38 deaths)  n validation = 455 (36 deaths)  **age at inclusion**: not reported  **resuscitation policy**: not reported  **inclusion criteria**: “a pre-existing data set” *No additional inclusion criteria were reported.*  mean birth weight:  training set = 1240g (5%-95% 634-2030)  validation set = 1330g (5%-95% 634-2100)  mean gestational age:  training set = 29w (5%-95% 25-33)  validation set = 29w (5%-95% 24-33)  **race**: 78% white | **outcome**: mortality within the first 28d of life  **mortality**: 8.3%  **time of prediction**: “within a few hours after birth”  **purpose**: “Using an ANN for admission data on preterm neonates we aimed to: predict the individual neonatal mortality risk of preterm neonates from their admission data; compare the net’s performance with that of a logistic regression model; and characterize survivors despite a high predicted individual mortality risk, and non-survivors despite a low predicted individual mortality risk.” | **model**: logistic regression, neural network  linearity: implies linear regression, neural network is nonlinear  *final model not reported*  *Final variables:*  gestational age  birth weight  condition on admission vitally endangered and condition on admission stable/unstable  inborn  capillary pH <7.10  temp on admission <35.5  emergency delivery  10 minute Apgar <5  5 minute Apgar <3  congenital malformation  1 minute Apgar  **validation**: temporally separate sample | Regression:  AUC = 0.917 (SD 0.017)  ANN complete input set:  AUC = 0.954 (SD 0.015)  skip gestational age:  AUC = 0.924 (SD 0.015)  skip birthweight:  AUC = 0.928 (SD 0.017)  skip condition on admission vitally endangered and condition on admission fair  AUC = 0.929 (SD 0.015)  skip condition on admission stable and condition on admission fair AUC = 0.930 (0.015)  skip inborn:  AUC = 0.936 (SD 0.016)  skip capilliary pH < 7.10 :  AUC = 0.943 (SD 0.015)  skip temperature on admission < 35.5°C:  AUC = 0.945 (SD 0.014)  skip condition on admission stable and condition on admission unstable  AUC = 0.945 (SD 0.014)  skip emergency delivery:  AUC = 0.946 (SD 0.013)  skip 10 minute Apgar score < 5 :  AUC = 0.947 (SD 0.013)  skip 5 minute Apgar score < 3 :  AUC = 0.947 (SD 0.013)  skip congential malformation:  AUC = 0.949 (SD 0.012)  skip 1 minute Apgar score:  AUC = 0.950 (SD 0.013)  skip gestational age and birthweight:  AUC = 0.881 (SD 0.029)  gestational age and birthweight alone:  AUC = 0.890 (SD 0.021)  **range**: (ANN complete set): <5% - <80% | “An artificial neural network trained on admission data can accurately predict the mortality risk for most preterm infants. However, the significant number of prediction failures renders it unsuitable for individual treatment decisions.” |
| Prediction of survival for preterm births by weight and gestational age: retrospective population based study.  Draper ES, Manktelow B, Field DJ, James D.  BMJ. 1999 Oct 23;319(7217):1093-7. | Trent health region, UK  1 January 1994 - 31 December 1997  **surfactant / antenatal steroids**:  [in contrast to this study, a previous similar study] was carried out before the introduction of... exogenous surfactant therapy and the widespread use of antenatal steroids. | 22-32 w gestation  n = 3760 (738 deaths, of which 271 were intrapartum stillbirths or delivery room deaths)  n = 3489 admitted to a NICU  **age at inclusion**: stillbirths included (onset of labor)  **resuscitation policy**: not reported, multicenter  **inclusion criteria**: all births and late fetal losses: all infants known to be alive at the onset of labor  excluded lethal congenital abnormalities  *summary statistics for birth weight and gestational age not reported*  **race**: European, Asian *(% not reported)* | **outcome**: survival status at discharge from the labor ward or neonatal unit  **mortality rate**:  7.2% stillbirths or delivery room deaths  19.6% overall  **time of prediction**: onset of labor (2 models)  admission to NICU (3 models)  **purpose**: We aimed to produce birth weight and gestational age specific survival graphs for a population of infants born between 22 and 32 weeks' gestation in the United Kingdom. | **model**: logistic regression  linearity: linear  *presented as weight/age charts*  *Final variables (all models)*:  birth weight  gestational age  race (White/Asian)  multiple births  gender  **validation**: not done | model singleton European infants known to be alive at the onset of labor:  H-L χ^2^=7.06, df=8, p=0.53  *Performance measure not given for other 4 models*  **range**: 2%-99% | “Birth weight and gestational age specific predicted survival graphs for preterm infants facilitate decision making for obstetricians, neonatologists, and parents and give a range of predicted survival for any given gestational age by estimated or actual birth weight. It is important that such graphs are produced for a geographically defined population so that they are representative and not biased towards the outcomes of particular centres. The production of such graphs in two stages allows for the changing pattern of survival from the start of the intrapartum period to the immediate period after admission for neonatal care. A continual process of updating needs to be in place to allow for improvements in survival of infants.” |
| SNAP-II and SNAPPE-II: Simplified newborn illness severity and mortality risk scores.  Richardson DK, Corcoran JD, Escobar GJ, Lee SK.  J Pediatr. 2001 Jan;138(1):92-100. | The Canadian NICU Network (17 NICUs)  January 1996 to October 1997  validation:  Kaiser Permanente, Northern California (6 NICUs)  August 1995 to March 1998  New England (7 NICUs)  October 1994 to June 1996  USA  **surfactant**: not reported  **antenatal steroids**: “We specifically did not include any measures of antenatal events, including corticosteroid treatment, because this may obscure the ill effects of improper treatment.” | All NICU admissions  subgroup <=1500g  n = 25429 (5387 VLBW)  n development = 10819 (2155 VLBW), 418 deaths  n validation = 14610, 368 deaths  n Canada = 5588 (1045 VLBW), 200 deaths  n California = 5530 (596 VLBW), 117 deaths  n New England = 3492 (1591 VLBW), 231 deaths  **age at inclusion**: NICU admission (data from deaths before 12h were included as partial scores)  **resuscitation policy**: not reported  **inclusion criteria**: all sequential admissions of infants of all birth weights during the designated study periods; we excluded: (1) babies released to the regular nursery in <24 hours; (2) babies admitted to the neonatal intensive care unit at >48 hours of age or after having been discharged home; (3) moribund infants when an explicit physician decision not to provide life support was made at the time of NICU admission; and (4) babies who died in the delivery room.  *number of infants in each birth weight category at each location are reported in a table*  **race**: % not reported | **outcome**: in-hospital mortality  **mortality**: overall 3.9%, not reported separately for <1500g  **time of prediction**: 12h  **purpose**: performance review and bench marking | **model**: score based on logistic regression  linearity: tested linearity for some variables  constant ß=4.69   | MBP 20-29 mm Hg ß=0.88 | 9 | | --- | --- | | MBP <20 mm Hg ß=1.94 | 19 | | Lowest temperature 95°-96°F ß=0.81 | 8 | | Lowest temperature <95°F ß=1.55 | 15 | | PO_2_/FiO_2_ ratio 1.0-2.49 ß=0.49 | 5 | | PO_2_/FiO_2_ ratio 0.3-0.99 ß=1.57 | 16 | | PO_2_/FiO_2_ ratio <0.3 ß=2.80 | 28 | | Lowest serum pH 7.10-7.19 ß=0.71 | 7 | | Lowest serum pH <7.10 ß=1.57 | 16 | | Multiple seizures ß=1.87 | 19 | | Urine output 0.1-0.9 mL/kg/h ß=0.46 | 5 | | Urine output <0.1 mL/kg/h ß=1.82 | 18 | | Birth weight 750-999 g | 10 | | Birth weight <750 g | 17 | | Small for gestational age (<3rd percentile) | 12 | | Apgar score at 5 minutes <7 | 18 |   **validation**: split-sample and geographically separate samples | <1500g  development:  AUC = 0.85 (+/-0.01)  H-L p=0.90  r = 0.90  validation:  Canada:  AUC = 0.86 (+/-0.02)  H-L p=0.78  r = 0.91  California:  AUC = 0.86 (+/-0.02)  H-L p=0.11  r = 0.83  New England:  AUC = 0.88 (+/-0.01)  H-L p=0.42  r = 0.92  combined:  AUC = 0.85 (+/-0.01)  H-L χ^2^=3.25, df=7, p=0.86  r = 0.90  r = Pearson correlations between SNAP-PE and SNAPPE-II  *observed-to-expected ratios per SNAPPE-II score category are reported in a table*  **range**: 0.3%-64.4% | “In summary, we have derived and validated simplified, empirically weighted illness severity and mortality risk scores that are specific for neonatal intensive care and applicable to infants of all birth weights. These scores are simple, accurate, and robust across many populations. They are accurately calibrated to very large, recent international NICU cohorts and can be similarly re-calibrated over time. They should be incorporated into routine use for performance review and bench marking.” |
| Characterization of multiple organ dysfunction syndrome in very low birthweight infants: a new sequential scoring system.  Janota J, Stranák Z, Statecná B, Dohnalová A, Sípek A, Simák J.  Shock. 2001 May;15(5):348-52. | Institute for the Care of Mother and Child, Prague, Czech Republic  December 1995 to November 1998.  **surfactant**: not reported  **antenatal steroids**: not reported | birth weight under 1500g and GA less than 31 weeks  n = 142 (16 deaths by 28 days, 24 deaths before discharge)  **age at inclusion**: admission to NICU  **resuscitation policy**: not reported  **inclusion criteria**: excluded invariably lethal abnormalities  mean birth weight = 1074g (SD 289, range 480-1490)  mean gestational age= 28.7w (SD 2.3, range 22-30) | **outcome**: neonatal mortality (by day 28) and hospital mortality  **mortality**: 16.9%  **time of prediction**: day 1 and day 28  **purpose**: “to describe the process of increasing physiologic derangement in critically ill newborns. The NEOMOD score can provide an everyday information about the function of those organ systems that are mainly responsible for mortality. In addition, it may predict mortality and be used in comparing the severity of illness among groups of patients.” | **model**: score  linearity: n/a  each item rated as  0 (no dysfunction)  1 (moderate dysfunction)  2 (severe dysfunction)  central nervous system  hemocoagulation balance  respiratory system  gastrointestinal system  cardiovascular system  renal function  acid-base balance  **validation**: not done, external validation published separately in 2008 | NEOMOD on the first day of admission:  28-day mortality:  AUC = 0.79  hospital mortality:  AUC = 0.75  Worst NEOMOD in first 28 days of life, or prior to death if dead before 28 days:  28-day mortality:  AUC = 0.95  hospital mortality:  AUC = 0.91  Model?  H-L χ^2^=1.34, df=4, p=0.854  Sn = 63.6%  Sp = 95.8%  **range**: *not reported* | “In conclusion, the results of our study indicate that similar to critically ill adults, secondary multiple organ dysfunction can be described in a majority of critically ill VLBW infants. To our knowledge this is one of the first attempts of assessing and describing the presence of a multiple organ dysfunction in a group of preterm newborns hospitalized in a neonatal intensive care unit.” |
| Early predictors of mortality in very low birth weight neonates.  Gera T, Ramji S.  Indian Pediatr. 2001 Jun;38(6):596-602. | Maulana Azad Medical College, New Delhi, India  March – December 1998  **surfactant / antenatal steroids**: “Antenatal steroids were rarely used and surfactant was not used in any of the babies.” | 500-1500g  n = 115 (36 early neonatal deaths, 11 late neonatal deaths)  **age at inclusion**: live birth  **resuscitation policy**: babies with birth weight <750g or multiple malformations were not mechanically ventilated  **inclusion criteria**: all consecutive intramural live born infants  mean birth weight:  survivors = 1323g (SD 160)  deaths = 1055g (SD 243)  mean gestational age:  survivors = 33.8w (SD 2.9)  deaths = 30.4w (SD 3.0) | **outcome**: “early neonatal mortality” and “late neonatal mortality”  **mortality**: 40.9%  **time of prediction**: 12-24 h  **purpose**: “to determine the factors predictive of mortality within the first 12-24 hours of life in the Indian setting” | **model**: logistic regression  linearity: not reported  Model 1:  birth weight 0.6072  shock 2.6169  pH 0.5337  alveolar arterial oxygen gradient 0.0032  *gestation*  *Apgar at 1, 5, and 10 minutes*  *need for resuscitation at birth*  Model 2:  birth weight 0.7396  mechanical ventilation 3.5274  **validation**: not done | CRIB >1  Sn = 71.7%  Sp = 83.4%  birth weight <1200g, need for ventilation, shock  Sp = 100%  Sn = 17%  shock  Sp = 93%  Sn = 25.5%  need for ventilation  Sp = 95.5%  Sn = 46.8%  birth weight <1200g  Sn = 63.8%  Sp = 88.2%  **range**: *not reported* | “In conclusion it can be said that VLBW neonates, especially those with a birth weight less than 1200 grams, who are critically ill due to disturbances in the pulmonary and circulatory physiology have a very high risk of mortality. Interventions such as the need for ventilation may increase the burden of mortality contributed to by severe illness. It is therefore imperative that NICUs caring for large number of VLBW infants must maintain high standards of neonatal care with a continous audit of the care provided in order to salvage more of these VLBW infants.” |
| CRIB II: an update of the clinical risk index for babies score.  Parry G, Tucker J, Tarnow-Mordi W; UK Neonatal Staffing Study Collaborative Group.  Lancet. 2003 May 24;361(9371):1789-91. | 54 UK units march 1998 – April 1999  secondary analysis of the UK Neonatal Staffing Study  **surfactant**: investigated as a variable, percentage treated not reported  **antenatal steroids**: mentioned in discussion but not reported | <=32w GA  n development = 1886  n validation = 1065  = 3027 (240 deaths)  **age at inclusion**: NICU admission (stillbirths excluded)  **resuscitation policy** not reported  **inclusion criteria**: primary study excluded 34 deaths classified as due to lethal congenital abnormalities or metabolic disorders, complex cardiac surgery or organ transplant, or resuscitated stillbirths.  mean birth weight = 1355g (SD 443)  median GA = 30w (IQR 28-31) | **outcome**: death before hospital discharge  **mortality rate**: 7.9%  **time of prediction**: within 1 hour of birth  **purpose**: CRIB II provides a recalibrated and simplified scoring system, which avoids the potential problems associated with use of FiO2 and data up to 12 h after admission; we developed and assessed CRIB II for infants of 32 weeks’ gestation, with further data available up to 1 h after admission to neonatal intensive care, excluding FiO2 | **model**: logistic regression  linearity: not reported  *presented as a score calculated from charts*  *Final variables:*  birthweight  gestation  sex  admission temperature  base excess  **validation**: randomized by center | CRIBII (validation)  AUC = 0.92 (SE 0.01)  H-L χ^2^=4.30, df=8, p=0.829  Cox slope p=0.642  Cox constant p=0.141  CRIB  1988-90  H-L χ^2^=51.6, df=9, p<0.001  Cox slope p=0.231  Cox constant p=0.001  1993-4  H-L χ^2^=28.3, df=9, p=0.001  Cox slope p=0.504  Cox constant p <0.001  Gestation  1988-90  H-L χ^2^=334.0, df=9, p<0.001  Cox slope p<0.001  Cox constant p<0.001  1993-4  H-L χ^2^=104.9, df=9, p<0.001  Cox slope p<0.001  Cox constant p<0.001  Birthweight  1988-90  H-L χ^2^=197.0, df=10, p <0.001  Cox slope p<0.001  Cox constant p<0.001  1993-4  H-L χ^2^=95.8, df=10, p<0.001  Cox slope p<0.001  Cox constant p<0.001  Death or major cerebral abnormality  CRIB-II: AUC = 0.82  CRIB: AUC = 0.79  GA: AUC = 0.80  BW: AUC = 0.77  Draper grid: AUC= 0.80  **range**: *not reported* | “The clinical risk index for babies (CRIB) score is a risk- adjustment instrument widely used in neonatal intensive care. Its appropriateness with contemporary data has been questioned. We have examined these questions, developed a new five-item CRIB II score with data from a UK-wide sample of infants admitted to neonatal intensive care in 1998–99, and shown how mortality after neonatal intensive care has fallen in the past 12 years. CRIB II provides a recalibrated and simplified scoring system that avoids the potential problems of early treatment bias. A valid and simple method of risk- adjustment for neonatal intensive care is important to ensure accurate assessment of quality of care. Such assessments should be done in tandem with national audit systems for neonatal intensive care, incorporating measures of morbidity as well as mortality.” |
| A new score for predicting neonatal very low birth weight mortality risk in the NEOCOSUR South American Network.  Marshall G, Tapia JL, D'Apremont I, Grandi C, Barros C, Alegria A, Standen J, Panizza R, Roldan L, Musante G, Bancalari A, Bambaren E, Lacarruba J, Hubner ME, Fabres J, Decaro M, Mariani G, Kurlat I, Gonzalez A; Grupo Colaborativo NEOCOSUR.  J Perinatol. 2005 Sep;25(9):577-82. | 16 Neonatal del Cono Sur Network participating centers from Argentina, Chile, Paraguay, Peru, and Uruguay  October 1, 2000 to May 30, 2003  **surfactant**: not reported  **antenatal steroids**: used in 74% of survivors and 53% of nonsurvivors | 500-1500g  n = 1801 (479 deaths)  n development = 1351  n validation = 450  **age at inclusion**: before NICU admission  **resuscitation policy**: not reported  **inclusion criteria**: born in participating centers; delivery room deaths included  mean birth weight = 1081g  mean GA = 29.2w | **outcome**: in-hospital mortality  **mortality**: 26.6%  **time of prediction**: before NICU admission  **purpose**: “The purpose of this study was to develop a neonatal mortality risk score for very low birth weight infants based on variables present at birth, before neonatal intensive care unit admission in a multicenter South American Network with diverse mortality rates and resources.” | **model**: logistic regression; “all variables... showed linear effect”  birth weight -0.331  gestational age -0.132  1 minute Apgar -0.265  ALT congenital malformation 3.419  antenatal steroid administration -0.302  female gender -0.474  constant = 8.378  **validation**: randomized sample | birth weight  AUC = 0.79  gestational age  AUC = 0.77  1 min Apgar  AUC = 0.77  NEOCOSUR  model  AUC = 0.88  test  AUC = 0.84  total  AUC = 0.87  H-L χ^2^=11.9, df=8, p=0.85  CRIB  AUC = 0.79  NICHD  AUC = 0.83  **range**: *not reported* | “We conclude that this new and relatively simple neonatal VLBW infant mortality risk score has good predictive performance in a multicenter South American population, and is an important tool for comparison purposes among NICUs. Based on its simplicity and good performance in a diverse population setting, we speculate that this risk score may prove to be a better model for application in developing countries” |
| Prenatal predictors of mortality in very preterm infants cared for in the Australian and New Zealand Neonatal Network.  Evans N, Hutchinson J, Simpson JM, Donoghue D, Darlow B, Henderson-Smart D.  Arch Dis Child Fetal Neonatal Ed. 2007 Jan;92(1):F34-40. | Australia & NZ  Australia and New Zealand Neonatal Network (ANZNN) database (98.8% of live births before 29 weeks and 92% of live births before 32 weeks)  1998-9 (development) and  2000-1 (validation)  **surfactant**: not reported  **antenatal steroids**: ~15.6% did not receive antenatal steroids; ~3.9% missing data | gestational age <32w *or* birthweight <1500g  n = 11215  n development = 5713 (390 deaths)  n validation = 5898 (373 deaths)  **age at inclusion**: NICU admission  **resuscitation policy**: “most NICUs would admit these infants and mortality would be less affected by different resuscitation policies”  **inclusion criteria**: excluded infants with gestational age <=24w,  excluded delivery-room deaths  median birth weight = 1230 g (IQR 960-1504)  median gestational age = 29w (IQR 27-30)  **ethnicity**: Caucasian/other 81.9%, Asian 6.3%,  Indigenous Australian 0.61%, Maori 0.17%, Pacific Islander 0.21% (excluded due to missing data) | **outcome**: death before discharge from the first hospitalization (followed through interhospital transfers)  **mortality**: 6.8%  **time of prediction**: up to 1 min postpartum  **purpose**: case mix adjustment | **model**: logistic regression; “the relationship is not  linear...”  intercept = -4.8802  GA  31w (0: reference)  30w (0.6075)  29w (1.1168)  28w (1.5269)  27w (2.2485)  26w (2.8459)  25w (3.4715)  Weight for GA centile group  97+ (0.3283)  75-<97 (-0.1767)  25-<75 (0: ref.)  3-<25 (0.7534)  <3 (2.0865)  Male sex (0.4359)  Hypertension in pregnancy (-0.7671)  **validation**: temporally separate validation set | development set:  AUC = 0.82  H-L goodness of fit statistic = 2.19, df=7, p=0.95  validation set:  AUC = 0.83  H-L goodness of fit statistic = 4.35, df=8, p=0.82  gestational age alone  AUC = 0.787  **range**: *not reported* | “In conclusion, this paper has described a simple, stable and accurate model using perinatal variables for prediction of mortality in very preterm infants. By using gestation rather than birth weight as the main indicator of maturity, these data show that weight for gestational age is an independent risk factor for mortality. This model will be a useful tool to control for case mix in the comparison of inter-NICU outcome.” |
| Predictors of death or bronchopulmonary dysplasia in preterm infants with respiratory failure  Ambalavanan N, Van Meurs KP, Perritt R, Carlo WA, Ehrenkranz RA, Stevenson DK, Lemons JA, Poole WK, Higgins RD  J Perinatol. 2008 Jun;28(6):420-6. | secondary analysis of Inhaled Nitric Oxide for Premature Infants with Severe Respiratory Failure trial  October 1, 2000 – May 20, 2003  USA  **surfactant**: cohort had been treated with surfactant at birth (4h before inclusion)  **antenatal steroids**: about 70% of cohort received antenatal steroids | birth weight 401-1500g  n = 420 (76-78% mortality or bronchopulmonary dysplasia (BPD); 44-52% mortality)  **age at inclusion**: 4h **resuscitation policy**: not reported.  **inclusion criteria**: subpopulation: severe respiratory failure >4h after treatment with surfactant  mean birth weight = 840g  mean GA = 26w (SD 2)  **race**: white (45.5%), black (35.0%), Hispanic (16.2%), other (3.3%)[[1]](#footnote-2) | **outcome**: death or BPD before discharge or within 365 days of start of trial  **mortality**: 52%  **time of prediction**: not specified, uses data available by 4h after birth  **purpose**: identify variables that predict death/BPD in preterm infants with severe respiratory failure; suitable for risk stratification but not for decisions regarding withdrawal of support | **model**: CART model  linearity: not reported  *model presented as a classification tree*  **validation**: not done | CART:  at 95% Sn:  PPV = 82%  NPV = 65%  Sp = 32%  regression:  full data set:  c statistic = .75  treatment (iNO):  c statistic = .84  control (no iNO):  c statistic = .76  **range**: *not reported* | “The major factors associated with death/BPD were an increased severity of respiratory failure, lower birth weight, male gender and outborn status, but not the magnitude of initial response to iNO.” |
| Predictors of mortality in very low birth weight neonates in India.  Basu S, Rathore P, Bhatia BD.  Singapore Med J. 2008 Jul;49(7):556-60. | University Hospital of the Institute of Medical Sciences, Banaras Hindu University, India  “over a period of 3 years”  **surfactant**: not reported  **antenatal steroids**: “Out of 260, only 46 mothers had received antenatal steroids, nine in group I and 37 in group II “ | <1500g  n = 260 (96 deaths)  no babies below 27w or 700g survived  **age at inclusion**: 12h  **resuscitation policy**: not reported  **inclusion criteria**: All newborns weighing less than 1,500 g admitted to NICU was included in the study. Exclusion criteria were: (1) neonates weighing less than 500 g and with gestational age less than 26 weeks; (2) presence of lethal congenital anomalies; and (3) death in the delivery room or within 12 hours of life.  *Birth weight and gestational age given in tables* | **outcome**: in-hospital death  **mortality**: 33.1%  **time of prediction**: 12h  **purpose**: The objective of the present study was to determine the predictors of mortality in VLBW neonates... The present study was undertaken to assess the predictors of mortality of VLBW infants and identify those neonates who require early referral and higher level care. | **model**: logistic regression  linearity: not reported  final model: maternal PV bleeding (1.3261)  apnoea (+ 3.159)  birth weight (0.0372)  gestational age (0.0627)  hypothermia (1.1323)  shock (+ 3.49)  **validation**: not done | predicted 65% of mortality  **range**: *not reported* | “Common antenatal and perinatal predictors of mortality in VLBW infants in India include maternal bleed, failure to administer antenatal steroids, low Apgar score, apnoea, extreme prematurity, neonatal septicaemia and shock.” |
| Perinatal factors associated with early deaths of preterm infants born in Brazilian Network on Neonatal Research centers.  Almeida MF, Guinsburg R, Martinez FE, Procianoy RS, Leone CR, Marba ST, Rugolo LM, Luz JH, Lopes JM  J Pediatr (Rio J). 2008 Jul-Aug;84(4):300-7. | eight public university tertiary hospitals in Brazil  June 2004 - May 2005  **surfactant**: 13/579 had surfactant in delivery room; 49/560 had at least one dose of surfactant  **antenatal steroids**: 54/579 had antenatal steroids | gestational age 23-33 weeks *and* birthweight 400-1,500 g  n = 579 (92 deaths)  **age at inclusion**: not specified  **resuscitation policy**: advanced resuscitation not used at all units  **inclusion criteria**: born alive, excluded malformations, excluded outborn/transfers  median birth weight = 1017 (SD 291)  median gestational age = 28.9w (SD 2.9) | **outcome**: death before 168h  **mortality**: 15.9%  **time of prediction**: 72h  **purpose**: evaluate perinatal factors associated with early neonatal death | **model**: logistic regression  linearity: not reported  *final model not reported*  *Final variables:*  controlled for SNAPPE-II  gestational age 23-27 weeks  maternal hypertension (absence)  5 min Apgar 0-6  RDS (present)  center of birth  **validation**: not done | H-L p=0.629  **range**: *not reported* | It can be concluded that the university units studied here, which are reference centers for the care of critically ill preterm patients, showed high rates of early neonatal mortality in comparison with developed countries standards, although these rates are much lower than average Brazilian data. A significant proportion of the risk factors associated with early neonatal mortality can be modified by interventions such as improving fetal vitality at birth and reducing the incidence and/or severity of respiratory distress syndrome by optimizing the use of antenatal corticosteroids and postnatal surfactant. Furthermore, the differences observed between the mortality rates at the eight units indicates that there is a need to identify possible best practices and adopt them uniformly throughout our country. |
| Simplified age-weight mortality risk classification for very low birth weight infants in low-resource settings.  Rosenberg RE, Ahmed S, Saha SK, Ahmed AS, Chowdhury MA, Law PA, Choi Y, Mullany LC, Tielsch JM, Katz J, Black RE, Santosham M, Darmstadt GL.  J Pediatr. 2008 Oct;153(4):519-24. Epub 2008 Jun 9. | Dhaka Special Care Nursery 1998-2003 (secondary analysis of trials on topical emollient therapy), Bangladesh  Cairo tertiary care NICU, Egypt  Nepal (secondary analysis of trial on chlorhexidine umbilical cord cleansing)  **surfactant**: not reported  **antenatal steroids**: not reported | <33 w  n = 467  n development = 428  324 Dhaka (148 survived)  54 Cairo (18 survived)  n validation = 39  Nepal (15 survived)  **age at inclusion**: NICU admission (excluded those “unlikely to live >48h”)  **resuscitation policy**: not reported (outborn infants only)  **inclusion criteria**: outborn infants admitted within 72h after birth  excluding congenital malformations and those judged unlikely to live beyond 48h of hospitalization  mean weight  Dhaka = 1157g (SD 197)  Cairo = 1300g (SD 202)  Nepal = 1219g (SD 210)  mean gestational age (average of methods)  Dhaka = 30.5w (SD 1.7)  Cairo = 31.0w (SD 1.5)  Nepal = 30.4w (SD 1.7) | **outcome**: death during hospital stay (Dhaka and Cairo) or at the end of the neonatal period (Nepal)  **mortality**:  overall 50.5%  Dhaka: 54%  Cairo: 66%  Nepal: 61%  **time of prediction**: not specified, variables available at birth  **purpose**: To identify a valid neonatal mortality risk prediction score feasible for use in developing countries. | **model**: logistic regression, presented as a score.  linearity: not reported  18 category SAWS:  male   | GA | BW |  | | --- | --- | --- | | <=29 | <=1000 | 15 | | <=29 | 1001-1250 | 12 | | <=29 | 1251-1500 | 12 | | 30-31 | <=1000 | 11 | | 30-31 | 1001-1250 | 4 | | 30-31 | 1251-1500 | 4 | | 32-33 | <=1000 | 10 | | 32-33 | 1001-1250 | 3 | | 32-33 | 1251-1500 | 3 |   female   | GA | BW |  | | --- | --- | --- | | <=29 | <=1000 | 13 | | <=29 | 1001-1250 | 10 | | <=29 | 1251-1500 | 10 | | 30-31 | <=1000 | 10 | | 30-31 | 1001-1250 | 1 | | 30-31 | 1251-1500 | 1 | | 32-33 | <=1000 | 9 | | 32-33 | 1001-1250 | 0 | | 32-33 | 1251-1500 | 0 |   2 category SAW:  <29 weeks or <1000g = high risk  >29 weeks and >1000g = moderate risk  **validation**: leave-one-out cross-validation of SAWS, and  validated on small geographically separate set (n = 39) | Weight categories only:  AUC = 0.6292 (SE 0.03)  cross-validation:  AUC = 0.4586  GA categories only:  AUC = 0.6493 (SE 0.03)  cross-validation:  AUC = 0.4756  GA + weight:  AUC = 0.6837 (SE 0.03)  cross-validation:  AUC = 0.5956  GA + weight + sex:  AUC = 0.7021 (SE 0.03)  cross-validation:  AUC= 0.6214  development (Dhaka and Cairo):  <32 weeks:  CRIB II  AUC = 0.622 (SE 0.03)  SAWS  AUC = 0.711 (SE 0.03)  CRIB-SAWS: p=.0058  SAW  AUC = 0.681 (SE 0.02)  SAWS-SAW: p=.0483  <33 weeks:  SAWS  AUC = 0.70 (SE 0.03)  SAW  AUC = 0.672 (SE 0.02)  SAWS-SAW: p=.100  29w and 1000g:  PPV: 92.3%  NPV: 39.9%  validation (Nepal):  <32 weeks:  CRIB II  AUC = 0.721 (SE 0.09)  SAWS  AUC = 0.698 (SE 0.09)  CRIB-SAWS: p=.5000  SAW  AUC = 0.69 (SE 0.08)  SAWS-SAW: p=.5480  <33 weeks:  SAWS  AUC = 0.679 (SE 0.09)  SAW  AUC = 0.670 (SE 0.075)  SAWS-SAW: p=.869  29w and 1000g:  PPV: 81.3%  NPV: 52.2%  **range**: SAWS 52.4%-96.5%  SAW: binary | “In conclusion, the SAW scoring system is a simple, valid, categorical MRS feasible for use in VLBW neonates in low-resource settings and builds on the model of CRIB II. The SAWS score has 3 criteria instead of CRIB II’s 5, and there are ultimately just 2 different categories in the SAW classification rather than 143 in CRIB II. Although the SAW classification did not have the high degree of internal validation of the CRIB II, the SAW MRS is much more feasible for a low-resource setting. The more detailed 18-category SAWS score may be used in specialized centers of developing countries for research purposes and benchmarking and to adjust parent expectations. The SAW binary discrimination between high- and moderate-risk categories would be most useful in community settings to guide referral decisions and promote care-seeking. Additional validity testing in other low-resource settings is recommended.” |
| The PREM score: a graphical tool for predicting survival in very preterm births.  Cole TJ, Hey EN, Richmond S.  Arch Dis Child Fetal Neonatal Ed. 2010 Jan; 95(1) : F14-9 | Northern (2000-3) and Trent (2000-4) regions,  UK  **surfactant**: not reported  **antenatal steroids**: not reported | 22–31 weeks gestation  n = 4838  Northern = 1456 (235 deaths + 22 stillborn)  Trent = 3382 (561 deaths + 68 stillborn)  **age at inclusion**: stillbirths included (onset of labor)  **resuscitation policy**: not reported (multicenter)  **inclusion criteria**: alive at onset of labor  excluding rapidly lethal malformations  *summary statistics for birth weight not reported*  median gestational age:  Northern = 29 w (IQR 26-30)  Trent = 29 w (IQR 27-30) | **outcome**: survival to term age  **mortality**:  overall 18.3%  17.6% Northern  18.5% Trent  **time of prediction**: prenatal (1 model)  birth (2 models)  **purpose**: to inform management and improve communication with parents | **model**: logistic regression; quadratic effect of gestational age, nonlinear effect of birthweight z-score  *3 models presented as isosurv graphs*  *Final variables:*  gestation  birthweight z score  base deficit  **validation**: not done, although birth model was initially developed on Northern population and refitted for Trent, the populations were then combined. | birth:  Northern:  AUC = 0.879  H-L p=0.3  Trent:  AUC = 0.887  H-L p=0.5  combined:  AUC = 0.882  H-L p=0.2  birth + base def:  AUC = 0.890  H-L p=0.8  birth + base def -BW:  AUC = 0.889  H-L p=0.8  prenatal:  AUC = 0.882  H-L p=0.2  **range**: 1%-98% | “We provide a logistic regression model and a graphical summary of the impact of gestation and birth size on survival in very preterm infants, which we believe should be useful for education, management, case mix adjustment and the interpretation of trends over time.” |
| Determinants of survival in very low birth weight neonates in a public sector hospital in Johannesburg.  Ballot DE, Chirwa TF, Cooper PA.  BMC Pediatr. 2010 May 6;10(1):30. | Charlotte Maxeke  Johannesburg Academic Hospital, Johannesburg, South Africa  1 July 2006 - 31 June 2007  **surfactant**:  given 90/474  not given 384/474  “Surfactant therapy was only given as rescue therapy to babies on ventilatory support... A second dose of surfactant could be given if the baby had responded to the initial dose and then deteriorated again.”  **antenatal steroids**:  given 173/474  not given 301/474 | <1501g birth weight  n = 448 (114 deaths)  **age at inclusion**: admission to NICU  **resuscitation policy**: ventilatory support only given to babies >900g  **inclusion criteria**: inborn and outborn  admitted to the neonatal unit within 24 hours of birth  mean birth weight = 1133.5g (95% CI 1111.9 – 1155.0)  mean gestational age = 29.9w  (95% CI 29.6-30.l) | **outcome**: mortality (to hospital discharge)  **mortality**: 25.4%  **time of prediction**: not reported; includes data from entire neonatal period  **purpose**: “The purpose of this study was to review the survival to hospital discharge and morbidity of VLBW infants at Charlotte Maxeke Johannesburg Academic Hospital (CMJAH), a busy neonatal unit in a developing country.” | **model**: logistic regression  linearity: not reported  *final model not reported*  *Final variables:*  birth weight  gender  resuscitation at birth  born before arrival  hypotension  definite NEC  provision of NCPAP  **validation**: not done | “The model predicted mortality correctly in 87% of cases. Birth weight was the single most important predictor of mortality, correctly predicting mortality in 82.9% of cases.”  **range**: *not reported* | “Survival rates compare favourably with other developing countries, but can be improved; especially in infants < 1001 grams birth weight. Resources need to be allocated to preventing the birth of VLBW babies outside hospital, early neonatal resuscitation, provision of NCPAP and prevention of NEC.” |

| ELGA/BW | | | | | | |
| --- | --- | --- | --- | --- | --- | --- |
| Study | setting, surfactant, and antenatal steroids | population, age at inclusion | outcome, time of prediction, purpose | model and validation | performance | conclusion |
| Survival of infants born at 24 to 28 weeks' gestation.  Herschel M, Kennedy JL Jr, Kayne HL, Henry M, Cetrulo CL.  Obstet Gynecol. 1982 Aug;60(2):154-8. | St Margaret's Hospital for Women  Boston, MA, USA  1 Jan 1977 – 31 Dec 1980  **surfactant**: not reported  **antenatal steroids**: it was the authors' policy to give betamethasone twice, 24h apart, to women at risk for premature delivery from 27 to 34w gestation. 68/135 received antenatal steroids, 11/73 infants prior to 27w.  **race:** 12% black / Hispanic (11/15 survived), 88% white (57/121 survived, NS) | 24-28 w GA  n = 135 (68 deaths)  **age at inclusion**: live birth  **resuscitation policy**: “all infants received oxygen, most infants >26w were ventilated if indicated and none at 24w were ventilated.”  **inclusion criteria**: inborn,  excluded 1 with congenital abnormalities incompatible with life, included 3 hydrops fetalis and 1 oligohydramniosis  excluded 17 stillbirths in this gestational age range  mean birth weight:  survived = 1050g (SD 198)  died = 897g (SD 217)  *summary statistics for gestational age not reported* | outcome: survival to discharge from nursery  **mortality**: 50.3%  **time of prediction**: not specified, variables are available at birth  **purpose**: “survival statistics are presented by gestational age...” | **model**: “multivariate analysis”  linearity: not reported  *final model not reported*  states that gestational age, maternal steroid administration, and resuscitation at birth were associated with survival with approximate ratios of 3:2:-1  **validation**: not done | “the survival outcome was correctly predicted in 80% of infants using this set of variables.”  **range**: *not reported* | “The data provide the obstetrician and neonatologist with survival statistics for infants of very early gestational age who were managed aggressively at gestations of at least 26 weeks. Because of the satisfactory survival for infants at 26 weeks and beyond, an aggressive approach to these fetuses is reasonable. There may be instances in which an aggressive approach to management of infants born before 26 weeks is justified, but the present study does not answer the question of whether this should be general policy. The data suggest that if infants managed in this way are going to die, approximately 50% will do so in the first 24 hours, approximately 80% within 1 week. Most infants of very early gestational age do not linger on only to die after weeks of intensive care. The long-term follow-up of these low birth weight survivors of the nursery is the subject of an ongoing investigation.” |
| Predicting survival of infants of birth weight less than 801 grams.  Zarfin J, Van Aerde J, Perlman M, Pape K, Chipman M.  Crit Care Med. 1986 Sep;14(9):768-72. | NICU of the Hospital for Sick Children, Toronto, Canada  Jan 1 1980 – 81 March 1984  **surfactant**: not reported  **antenatal steroids**: not reported | < 801g birth weight  n = 106 (58 deaths)  **age at inclusion**: 8h  **resuscitation policy**: not reported  **inclusion criteria**: outborn;  admitted within 8h of birth  excluded congenital abnormalities (26), excluded those who were moribund upon arrival (23), excluded infants who died before 8h (8)  *summary statistics for birth weight and gestational age not reported* | **outcome**: mortality between 24h – 28d  **mortality**: 51.7%  **time of prediction**: 8h, 16h, 24h  **purpose**: not specified | **model**: logistic regression  linearity: linear  *final model not reported*  *Final variables:*  8h:  birth weight  FIO_2_  16h and 24h:  temperature  pH  spontaneous breathing  **validation**: not done | cutoff: 0.50  correctly predicted to survive:  8h = 76%  16h = 79%  24h = 80%  correctly predicted to die:  8h = 77%  16h = 78%  24h = 77%  overall correct prediction:  8h = 76%  16h = 79%  24h = 79%  7/8 surviving infants with poor prognosis had severe morbidities; compared with 3/48 infants with >50% predicted survival  6/12 infants who were predicted to survive, but died, had treatment withdrawn.  **range**: 16%-99% | “Preliminary impressions arising from the observed relationship between survival against the odds and severe sequelae suggest that the above degree of accuracy may be enhanced by inclusion of late deaths and severe, irreversible handicap in the analysis of results. This relationship, however, needs to be established at long-term follow-up of this cohort of infants.” |
| Obstetric variables predicting survival of the immature newborn (less than or equal to 1000 gm): a five-year experience at a single perinatal center.  Amon E, Sibai BM, Anderson GD, Mabie WC.  Am J Obstet Gynecol. 1987 Jun;156(6):1380-9. | EH Crump Woman's Hospital and perinatal center  Memphis, TN, USA  5 year period ending 31 Dec 1985  **surfactant**: not reported  **antenatal steroids**: betamethasone was used in a nonstandard fashion since there were differing opinions among the subspecialists. | birth weight <1000g  n = 476 (192 survived)  **age at inclusion**: live birth  **resuscitation policy**: “A policy for resuscitation of all newborns was in effect, and intervention was taken on behalf of the fetus if GA >25w and estimated weight >600g.”  **inclusion criteria**: All live born, inborn infants, including delivery room deaths, congenital anomalies, infants who died after discharge to another unit. Infants weighing <500g were excluded if they died within 24h.  mean birth weight = 764g (156g)*?*  median birth weight = 771g (range 310-1000g)  mean gestational age = 26.0w (2.2)  median gestational age = 26w (range 22-33w)  **race**: black 76%, white 24% | **outcome**: survival: discharge home alive or to the morgue  **mortality**: 59.6%  **time of prediction**: not specified, includes variables up to 5 min after birth  **purpose**: “The purpose of this study was to provide an in-depth univariate and multivariate evaluation of the obstetric variables associated with neonatal survival of infants weighing <1000g.” | **model**: standardized canonic linear discriminant anlaysis  birth weight (0.56535)  5 minute Apgar (0.31603)  gestational age at delivery (0.31196)  decreasing cervical dilatation on admission (-0.25877)  female sex (0.22222)  a more recent study interval (0.21020)  race (0.20887)  **validation**: not done | survivors were accurately predicted in 76.2% and nonsurvivors in 69.3% of cases  **range**: *not reported* | *no conclusion section* |
| Viability, morbidity, and resource use among newborns of 501- to 800-g birth weight. National Institute of Child Health and Human Development Neonatal Research Network.  Tyson JE, Younes N, Verter J, Wright LL.  JAMA. 1996 Nov 27;276(20):1645-51. | 12- center National Institute of Child Health and Human Development Neonatal Research Network (USA)  January 1, 1994 - December 31, 1995  **surfactant**: “Surfactant therapy was used throughout the study period in all network centers.”  **antenatal steroids**: “Antenatal steroids were administered to 50% of mothers of 501- to 800-g infants.”  Of infants given MV (development set):  surfactant: 81%  antenatal steroids: 55% | 501-800g birth weight  n = 947 received mechanical ventilation (633 survived)  an additional 179 infants did not receive mechanical ventilation and were not included for model development  n development = 622  n validation = 325  **age at inclusion**: receiving mechanical ventilation  **resuscitation policy**: not reported (multicenter)  **inclusion criteria**: inborn;  excluded 3 with ambiguous genitalia, and 3 due to missing data  mean birth weight = 660g (SD 83)  mean gestational age = 24.8w (SD 2.0)  **race**: black 54%, white 31% (NS) | **outcome**: discharge to home  **mortality**: 33.5%  **time of prediction**: soon after birth  **purpose**: “To assess risk factors affecting viability and analyze the effects of mechanical ventilation (MV) on neonatal outcome and resource use among extremely premature infants” | **model**: logistic regression  linearity: not reported  *model presented as a table of probabilities*  **validation**: randomized sample | development:  AUC = 0.76  validation:  at cutoff 0.50%:  predicted to die: 48%  predicted to survive: 79%  (= 72% overall correct classification)  Sn = 43%  Sp = 85%  4 had a predicted probability of death >80%, 2 of these survived.  **range**: 35%-91% | “Although recommendations to initiate or forgo MV for extremely premature infants have often focused on 1 factor (birth weight or gestational age), multiple factors should be considered. Other factors being equal, our analyses support use of MV for females at a minimum birth weight approximately 100 g lower than that for males. The current diagnosis related group reimbursement system can be expected to compromise resources for 501- to 800-g infants who would benefit from MV. Such care entails considerable resource use, although the cost per lifeyear gained is likely to be considerably less than that for many adults given intensive care. Our findings can be used to facilitate more appropriate treatment decisions, determine adequate resources, and better inform the debate about the benefits and burdens of intensive care for extremely premature newborns.” |
| Outcome at 5 years of age of children 23 to 27 weeks' gestation: refining the prognosis.  Doyle LW; Victorian Infant Collaborative Study Group  Pediatrics. 2001 Jul;108(1):134-41. | Victoria, Australia  2 year period beginning January 1, 1991  includes 3 level III centers and surrounding level II centers  **surfactant**:  49.1% deaths. 43.1% survivors  **antenatal steroids**: 55.5% deaths,  71.1% survivors | 23 to 27 weeks  (no survivors born before 23 weeks)  n = 401 (176 deaths)  **age at inclusion**: live birth  **resuscitation policy**: not reported (multicenter)  **inclusion criteria**: A geographically determined cohort, live births of 23 to 27 weeks’ gestational age  mean birth weight:  died = 802g (SD 177)  survived = 891g (SD 176)  mean gestational age:  died = 25.2w (SD 1.2)  survived = 25.9w (SD 1.1)  **race**: Asian mother 7.2% | **outcome**: death or disability by 5 years of age  **mortality**: 43.9%  **time of prediction**: not reported, but divides data into 4 sets: obstetric, birth, >day7, and >day 28  **purpose**: To determine the changes with postnatal age for survival, with and without major sensorineural disability, to 5 years of age in very preterm infants and to contrast their prognosis with normal birth weight (NBW) control participants. | **model**: forward conditional logistic regression  linearity: not reported  *final model not reported*  *Final variables:*  increasing maturity  antenatal corticosteroid therapy  being a multiple birth  being female  not being SGA  **validation**: not done | observed and predicted rates of survival to 5 years for very preterm children offered intensive care related to common combinations of perinatal variables, averaged over all gestational ages: Hosmer-Lemeshow goodness-of-fit test, p=0.75  observed and predicted rates of survival free of major sensorineural disability for very preterm children discharged home related to the number of adverse variables: Hosmer-Lemeshow goodness-of-fit test, p=0.71  **range**: *not reported, although several examples are given* | “The prognosis for very preterm children varies with the place of birth (level III perinatal center or not), the attitude of both obstetricians and pediatricians toward care and hence the interventions they use, gestational age, postnatal age, and then later comorbidities. Because the prognosis changes substantially with postnatal age, counseling of families should be repeated at intervals, and the advice offered should vary with perinatal events.” |
| Comparison of the prediction of extremely low birth weight neonatal mortality by regression analysis and by neural networks.  Ambalavanan N, Carlo WA.  Early Hum Dev. 2001 Dec;65(2):123-37. | University of Alabama at Birmingham Regional NICU, USA  Jan 1990 – Dec 1996  **surfactant**: mentioned in introduction, but number treated not reported  **antenatal steroids**: “At least one dose of antenatal steroids had been received by 23% of the training set and by 25% of the test set”  **race**: approx. 37% Caucasian | birth weight <1000g  n = 810  n development = 502 (34% mortality)  n validation = 59  n test = 249 (33% mortality)  **age at inclusion**: admission to NICU  **resuscitation policy**: neonates >500g were aggressively resuscitated unless major malformations were present  **inclusion criteria**:  inborn and outborn; excluded delivery room deaths  mean birth weight:  training set = 749g (SE 8)  test set = 738g (SE 12)  mean gestational age:  training set = 25.8w (SE 0.1)  test set = 25.5w (SE 0.2) | **outcome**: mortality  **mortality**: 34%  **time of prediction**: within 6h of birth  **purpose**:“To compare the prediction of mortality in individual extremely low birth weight (ELBW) neonates by regression analysis and by artificial neural networks”  In the conclusion, the authors state that the accuracy is not sufficient for individual clinical prediction although it is sufficient for case mix adjustment | **model**: logistic regression and neural network  linearity: implies linear regression, neural networks are non-linear  regression with limited data set:  Logit P (survival 0, death 1) = 7.420(0.00595*Birthweight in grams)  (0.141*Apgar 50)( 0.672*Race W0 B1)(0.123*Gest Age in weeks) (0.725*Antenatal  Steroids 0 or 1)+(0.811*Multiple births 1, 2, 3, etc.)(0.494*RDS 0 or 1)  **validation**: randomized sample | cutoff 80% sens:  Neural network with full data set:  AUC = 0.83 (SE 0.03)  Sp = 69%  PPV = 56%  NPV = 88%  Regression with full data set:  AUC = 0.82 (SE 0.03)  Sp = 72%  PPV = 58%  NPV = 88%  cumulative r^2^ = 0.36  Neural network with limited data set:  AUC = 0.87 (SE 0.03)  Sp = 85%  PPV = 72%  NPV = 90%  Regression with limited data set:  AUC = 0.87 (SE 0.03)  Sp = 85%  PPV = 72%  NPV = 90%  + 16 models excluding one variable  **range**: *not reported* | “In summary, although both neural networks and logistic regression are able to predict mortality in the individual ELBW neonate with reasonable accuracy, they are still not perfect models, and while the degree of accuracy is sufficient for purposes such as quality assurance (e.g. investigation into causes of death when a neonate who is predicted to live dies unexpectedly, or for the comparison of two or more NICUs with differing death rates although with apparently similar patient populations), social and cultural considerations dictate that the use of these techniques for prediction and decision making in the individual neonate have extremely high positive and negative predictive values. Such an improvement in prediction performance may require more discriminative clinical information and better diagnostic criteria in clinical records rather than improved modeling techniques. Enhanced awareness of the major determinants of ELBW mortality and/or morbidity, combined with an ability to collect and analyze large amounts of clinical or laboratory data, may improve our ability to aid with selective early intervention and ethical decision making. Until then, birthweight and the Apgar score at 5 min would continue to remain the best clinical predictors of ELBW mortality.” |
| Factors affecting survival in infants weighing 750 g or less.  Locatelli A, Roncaglia N, Andreotti C, Doria V, Doni D, Pezzullo JC, Ghidini A.  Eur J Obstet Gynecol Reprod Biol. 2005 Nov 1;123(1):52-5. | San Gerardo Hospital, University of Milano-Bicocca, Italy  January 1998 - December 2002  **surfactant / antenatal steroids**: “During the study period, the overall obstetric and neonatal management of viable babies was aggressive starting at 24 weeks, and it included the use of antenatal corticosteroids... Neonatal care included... natural surfactant on intubation”  Antenatal steroids given for 87% of survivors, 59% of deaths. | <750g birth weight and  <34w GA  n = 59 (29 deaths)  placental exam possible in 53  **age at inclusion**: live birth  **resuscitation policy**: every live-born infant starting at 24w was resuscitated if indicated  **inclusion criteria**: non-malformed, live-born singleton, inborn  mean birth weight:  survivors = 650g (SD 82)  nonsurvivors = 575g (SD 91)  mean gestational age:  survivors = 27.3w (SD 1.4)  nonsurvivors = 25.4w (SD 1.9) | **outcome**: survival to discharge  **mortality**: 49.2%  **time of prediction**: not specified, variables available at birth  **purpose**: “Our study aims at identifying independent perinatal predictors of neonatal survival at a single institution with the willingness of obstetrician and neonatologist to optimize perinatal survival.” | **model**: logistic regression  linearity: not reported  gestational age 0.751  birth weight 0.018  female gender 1.994  Apgar score 5 min 0.903  antenatal steroids 2.215  **validation**: not done | explained: 69% of neonatal survival (R^2^)  Sn = 87%  Sp = 76%  PPV = 79%  NPV = 85%  **range**: *not reported* | “The predictors of survival among micropremies are the same as those reported for older preterm neonates. The type of preterm delivery (spontaneous versus indicated) and placental pathology do not independently affect survival.” |
| Prediction of death for extremely low birth weight neonates.  Ambalavanan N, Carlo WA, Bobashev G, Mathias E, Liu B, Poole K, Fanaroff AA, Stoll BJ, Ehrenkranz R, Wright LL; National Institute of Child Health and Human Development Neonatal Research Network.  Pediatrics. 2005 Dec;116(6):1367-73. | 19 centers of the Neonatal Research Network  USA  Jan 1 1998 – April 9 2003  **surfactant / antenatal steroids**: “However, clinical practices (prenatal steroid use and surfactant use) have changed significantly [since 1993]”  Prenatal steroid use is a significant variable in this analysis. | 401-1000g  n = 8608 but subsequent models censored by prior deaths and missing data:  A (limited prenatal):  n = 8608  n training = 6026  n test = 2582  B (full prenatal):  n = 8343  n training = 5840  n test = 2503  C (first 5 min):  n = 8343  n training = 5840  n test = 2503  14.3% mortality by 24h  D (first 24 h):  n = 6780  n training = 4746  n test = 2034  22.4% mortality by 7d  E (first 7 d):  n = 5973  n training = 4181  n test = 1792  **age at inclusion**:  live birth (3 models)  24h (1 model)  7 days (1 model)  **resuscitation policy**: information was not available  **inclusion criteria**: all live-born infants admitted to the 19 centers  at scenario A:  mean birth weight = 735g (SD 158)  mean gestational age = 25.5w (SD 2.3)  **race**: non-Hispanic black 43% | **outcome**: death  **mortality**: 35% by discharge  **time of prediction**: 5 models:  A – prenatal  B – prenatal  C – birth  D – 24h  E – 7 days  **purpose**: not specified | **model**: logistic regression and neural network  linearity: implies linear regression, neural network is nonlinear  *regression coefficients reported for each variable in each model, weights for neural network not reported*  **validation**: randomized sample | regression:  model A:  AUC = 0.814 (SE 0.008)  H-L p<0.01  at 50% Sn:  Sp = 93%  PPV = 80%  NPV = 78%  at 90% Sn:  Sp = 35%  PPV = 43%  NPV = 87%  model B:  AUC = 0.802 (SE 0.007)  H-L p<0.01  at 50% Sn:  Sp = 93%  PPV = 80%  NPV = 78%  at 90% Sn:  Sp = 35%  PPV = 43%  NPV = 87%  model C:  AUC = 0.854 (SE 0.004)  H-L p<0.01  at 50% Sn:  Sp = 95%  PPV = 84%  NPV = 79%  at 90% Sn:  Sp = 55%  PPV = 51%  NPV = 93%  model D:  AUC = 0.804 (SE 0.014)  H-L p=0.12  at 50% Sn:  Sp = 89%  PPV = 58%  NPV = 85%  at 90% Sn:  Sp = 49%  PPV = 35%  NPV = 94%  model E:  AUC = 0.791 (SE 0.010)  H-L p=0.2  at 50% Sn:  Sp = 90%  PPV = 49%  NPV = 90%  at 90% Sn:  Sp = 43%  PPV = 25%  NPV = 96%  Neural network:  model A:  AUC = 0.816 (SE 0.007)  model B:  AUC = 0.804 (SE 0.006)  model C:  AUC = 0.844 (SE 0.005)  model D:  AUC = 0.714 (SE 0.11)  model E:  AUC = 0.729 (SE 0.044)  **range**: *not reported* | “Prediction of death is limited even with sophisticated statistical methods such as logistic regression and nonlinear modeling techniques such as neural networks. The difficulty of predicting death should be acknowledged in discussions with families and caregivers about decisions regarding initiation or continuation of care.” |
| Early prediction of poor outcome in extremely low birth weight infants by classification tree analysis.  Ambalavanan N, Baibergenova A, Carlo WA, Saigal S, Schmidt B, Thorpe KE; Trial of Indomethacin Prophylaxis in Preterms (TIPP) Investigators.  J Pediatr. 2006 Apr;148(4):438-444. | 32 centers in Canada, US, Australia, NZ, and Hong Kong  Jan 1996 – March 1998  secondary analysis of TIPP trial  **surfactant / antenatal steroids**: “after the use of antenatal steroids and postnatal surfactant had become standard practice...” Tests antenatal steroids given <>7d | 500-999g birth weight  n = 1036 (435 deaths)  n development = 784  n validation = 262  **age at inclusion**: only those who survived to 8d  stillbirths/resuscitation:  stillbirths (and other deaths <8d) excluded, **resuscitation policy** information not available  **inclusion criteria**: infants who were enrolled in the TIPP trial who survived beyond 8 days of age  excluded deaths in the first week (97), lost to follow up (13) or missing outcome data (46)  mean birth weight = 787g (SD 130)  mean gestational age = 26.1w (SD 1.9)  **ethnicity**: *% not reported* | **outcome**: combined outcome of death and neurodevelopmental impairment (NDI) at a corrected age of 18 months  **mortality**: 41.9%  **time of prediction**:  antenatal (2 models)  day 3 (2 models)  day 8 (2 models)  **purpose**: “To predict death or neurodevelopmental impairment in extremely low birth weight infants by classification trees with recursive partitioning and automatic selection of optimal cut points... the aim is not to predict later NDI/death at the time of 1 week, but to determine whether we need to wait until 1 week of age for such prognostication.” | **model**:  classification tree  *model presented as a classification tree*  **validation**: randomized sample | 3 Terminal nodes  Antenatal  Sn = 0.49 (0.40-0.59)  Sp = 0.71 (0.63-0.78)  PPV = 0.58 (0.47-0.67)  NPV = 0.64 (0.56-0.71)  accuracy = 0.61 (0.55-0.67)  **range**: 27%-52%  3 days  Sn = 0.31 (0.23-0.40)  Sp = 0.86 (0.80-0.91)  PPV = 0.64 (0.50-0.77)  NPV = 0.61 (0.54-0.68)  accuracy = 0.62 (0.56-0.68)  **range:** 29%-65%  1 week  Sn = 0.34 (0.25-0.43)  Sp = 0.83 (0.76-0.89)  PPV = 0.61 (0.48-0.73)  NPV = 0.61 (0.54-0.68)  accuracy = 0.61 (0.55-0.67)  **range**: 29%-64%  5 Terminal nodes  Antenatal  Sn = 0.53 (0.44-0.63)  Sp = 0.69 (0.61-0.77)  PPV = 0.58 (0.48-0.67)  NPV = 0.65 (0.57-0.73)  accuracy = 0.62 (0.56-0.68)  **range**: 0%-56%  3 days  Sn = 0.31 (0.23-0.40)  Sp = 0.86 (0.80-0.91)  PPV = 0.64 (0.50-0.77)  NPV = 0.61 (0.54-0.68)  accuracy = 0.62 (0.56-0.68)  **range**: .06%-46%  1 week  Sn = 0.40 (0.31-0.49)  Sp = 0.79 (0.71-0.85)  PPV = 0.60 (0.48-0.71)  NPV = 0.62 (0.55-0.69)  accuracy = 0.61 (0.55-0.67)  **range**: 27%-83% | “The ability to predict long-term morbidity/death in extremely low birth weight infants does not improve significantly over the first week of life. Effects of different variables depend on age.” |
| Apgar score predicts short-term outcome in infants born at 25 gestational weeks.  Forsblad K, Källén K, Marsál K, Hellström-Westas L.  Acta Paediatr. 2007 Feb;96(2):166-71. | Nine neonatal units in the southern region of Sweden (1 level 3 and 7 level 1&2 )  1995-2001  **surfactant**: not reported  **antenatal steroids**: ...the perinatal database did not contain [antenatal corticosteroid treatment]. However [in a similar cohort] around 80% received antenatal steroids | 25 weeks GA  n = 92 (20 deaths, 26 death or severe brain damage)  **age at inclusion**: live birth  **resuscitation policy**: actively resuscitated at 25w  **inclusion criteria**: excluded stillbirths, included congenital malformations (3)  *mean birth weights given for subgroups* | **outcome**: survival at 180 postnatal days, and survival without severe brain damage at 180 days  **mortality**: 21%  **time of prediction**:  birth +1 min  birth +5 min  birth +10 min  CRIB (12h)  **purpose**: The aim of the present study was to investigate whether the Apgar score and other early available postnatal vari- ables including the CRIB, are associated with short term outcome in extremely preterm infants born at 25 gestational weeks who are subjected to active delivery management and resuscitation. | **model**: logistic regression  survival without severe brain damage (1 min):  1 min Apgar score:  constant β = -0.357  1 min Apgar score β = 0.267117  survival without severe brain damage, best model (5 min):  constant β = -0.452, Apgar score at 5 min β = 0.415, multiple vs singleton β = -1.018  survival without severe brain damage using CRIB:  constant β = -0.646, Apgar score at 5 min β = 0.630, CRIB score β = -0.350  survival, best model (5 min):  constant β = -0.323, Apgar score at 5 min β = 0.408, multiple vs singleton β = -0.791  **validation**: not done | survival without severe brain damage (1 min):  p = 0.01636855  H-L χ^2^=12.28, df=8, p=0.14  **range**: (graph) approx. 50%-90%  survival without severe brain damage. best model (5 min):  p=0.00796  H-L χ^2^=4.66, df=8, p=0.793  **range**: (graph) approx. 15%-90%  survival, best model (5 min):  p=0.0015.  H-L χ^2^=9.04, df=8 p=0.339  **range**: (graph) approx. 45%-95%  (10 min Apgar “was no better”)  survival without severe brain damage using CRIB:  p=2.0 x 10^-5^  H-L χ^2^=8.93, df=8, p=0.348  **range**: (graph) approx. 5%-95% | “Apgar score predicts short-term outcome in extremely preterm infants at 25 gestational weeks. The precision for prediction of outcome increases when Apgar score is combined with CRIB.” |
| Intensive care for extreme prematurity – moving beyond gestational age.  Tyson JE, Parikh NA, Langer J, Green C, Higgins RD; National Institute of Child Health and Human Development Neonatal Research Network.  N Engl J Med. 2008 Apr 17;358(16):1672-81. | 19 centers of the Neonatal Research Network of the National Institute  of Child Health and Human Development USA  January 1, 1998 - December 31, 2003  **surfactant**: not reported  **antenatal steroids**: 80% for infants whom mechanical ventilation was initiated, 71% overall | 22-25 completed weeks *and* 401-1000g BW  n = 3702 (38% mortality before discharge; 42% mortality by 18-22 mos)  **age at inclusion**: initiation of mechanical ventilation / admission to NICU  **resuscitation policy**: not reported (multicenter)  **inclusion criteria**: excluded infants with a major anomaly  excluded infants with a birth weight >97^th^ centile for gestational age (indicating that GA was underestimated)  excluded 31 infants who survived without mechanical ventilation  excluded infants who did not receive intensive care  mean birthweight:  all infants = 648g (SD 124)  intensive care = 670g (SD 118)  mean gestational age:  all infants = 23.9w (SD 0.99)  intensive care =24.2w (SD 0.82)  **race/ethnicity**: black 45%, white 35%, Hispanic 17% | **outcome**: death at 18 mos of age  **mortality**: 42%  **time of prediction**: at the time of decision to initiate mechanical ventilation  **purpose**: “To facilitate more informed and better justified decisions...to relate gestational age and other risk factors assessable at or before birth to the likelihood of death or adverse neurodevelopmental outcomes.... we adopted the perspective of a physician deciding whether to initiate mechanical ventilation” | **model[[2]](#footnote-3)**: logistic regression  linearity: not reported  death at 18m:  p(Death) = 1 / { 1 + e [ - ( 3.3900 + -0.005113 x Birth Weight (g) + 1.5741 (if GA*=22) + 0.9598 (if GA=23) + 0.4721 (if GA=24) + -0.4454 (if Female) + -0.2582 (if Singleton) -0.6062 (if antenatal steroids)) ] }  death or profound impairment:  p(Death/PI) = 1 / { 1 + e [ - ( 4.0874 + -0.004941 x Birth Weight (g) +  1.6552 (if GA=22) + 0.9644 (if GA=23) + 0.4195 (if GA=24) +  -0.5898 (if Female) + -0.2751 (if Singleton) + -0.6233 (if antenatal steroids)) ] }  **validation**: bootstrap | death at 18m:  gestational age alone:  AUC = 0.709 (95% CI 0.692–0.726)  new model:  AUC = 0.753 (95% CI 0.737–0.769)  death or profound impairment:  gestational age alone:  AUC = 0.704 (95% CI 0.686–0.721)  new model:  AUC = 0.751 (95% CI 0.735–0.767)  **range**: *not reported* | “The likelihood of a favorable outcome with intensive care can be better estimated by consideration of four factors in addition to gestational age: sex, exposure or nonexposure to antenatal corticosteroids, whether single or multiple birth, and birth weight.” |
| Short-term outcome predictors in infants born at 23-24 gestational weeks.  Forsblad K, Källén K, Marsál K, Hellström-Westas L.  Acta Paediatr. 2008 May;97(5):551-6. | Swedish national birth register  January 2000 – December 2002  **surfactant**: not reported  **antenatal steroids**: not recorded in the source database | 23 and 24 w GA  n = 156  23w0d to 23w6d  n = 57 (23 survived, 3 with severe brain damage)  24w0d to 24w6d  n = 99 (49 survived, 10 with severe brain damage)  **age at inclusion**: live birth  **resuscitation policy**: not known; about 40% of 23-week infants were probably not resuscitated and were considered “stillborn”  **inclusion criteria**: all live born infants  mean birth weight:  23w = 563g (SD 79)  24w = 659g (SD 125) | **outcome**: survival at 180 postnatal days, and survival without a neonatal diagnosis implying severe brain damage  **mortality**:  overall: 53.8%  23w: 59.6%  24w: 50.5%  **time of prediction**: birth (within 5 minutes)  **purpose**: “to evaluate whether the Apgar score and other factors that are present and known at birth are predictive of outcome in extremely preterm infants born at 23 and 24 gestational weeks.” | **model**: multiple logistic regression analyses  23 weeks (survival):  Constant β = 0.251  Apgar score at 5 min  β = 0.467.  Multiple births versus singleton birth: β = – 2.162  24 weeks (survival):  constant β␤ = 0.221.  Apgar score at 5 min β = 0.326  Multiple births versus singleton birth: β = –1.582  24 weeks (survival without severe brain damage):  constant β␤ = -0.524.  Apgar score at 5 min β = 0.274  Multiple births versus singleton birth: β = –1.160  **validation**: not done | 23 weeks (survival):  p = 0.0005.  H-L χ^2^=11.55, df=8, p=0.17  **range**: (graph) approx. 5%-90%  24 weeks (survival):  p = 0.00006  H-L χ^2^=6.23, df=8, p=0.622  **range**: (graph) approx. 5%-90%  24 weeks (survival without severe brain damage):  p = 0.00243  H-L χ^2^=6.52, df=8, p=0.590  **range**: (graph) approx. 10%-70% | “The 5-min Apgar score is associated with short-term outcome in live-born infants at 23–24 gestational weeks. The association is stronger for infants born at 24 weeks of gestation. ” |
| Unimpaired outcomes for extremely low birth weight infants at 18 to 22 months.  Gargus RA, Vohr BR, Tyson JE, High P, Higgins RD, Wrage LA, Poole K.  Pediatrics. 2009 Jul;124(1):112-21. | 19 National Institute of Child Health and Human Development Neonatal Research Network centers  USA  January 1, 1998 - December 31, 2001  **surfactant**: not reported  **antenatal steroids**: not reported | 401-1000g  n = 5250 (2100 deaths: 886 within 12h, 1132 12h-discharge, and 82 after discharge)  645 were lost to follow-up, 195 had incomplete assessments, both were excluded  n model 3 = 3232  **age at inclusion**:  live birth (2 models)  discharge (1 model)  **resuscitation policy**: not reported  **inclusion criteria**: inborn, including delivery room deaths, excluding those lost to follow-up  *mean birth weights and gestational age shown for each of 4 subgroups*  **race**: black 45.7%, white 36.9%, hispanic 14.4%, other 3.0% (in cluster) | **outcome**: 2-level outcome of survival to 18 months or death; 3-level outcome of unimpaired survival, mildly impaired survival, or impaired survival or death  **mortality**: 34.4%  **time of prediction**: model 1 and 2: birth, model 3: end of neonatal period (discharge)  **purpose**: “the objectives of this study were to determine the unimpaired survival rate among ELBW infants and to identify the associated perinatal, neonatal, and socioenvironmental characteristics at corrected age of 18 to 22 months.” | **model**: logistic regression  linearity: Second order terms for continuous variables were also considered in the model, to test for a nonlinear relationship...  *final model not reported*  *Final variables*  model 1: mortality at 18m:  maternal age  married  white  Female  birth weight  singleton  SGA  Prenatal care  prenatal steroid use  cesarean section  model 2: unimpaired or mildly impaired vs NDI or death at 18m:  maternal age  married  white  female  birth weight  singleton  SGA  prenatal care  prenatal steroid use  cesarean section  model 3: unimpaired or mildly impaired vs NDI or death at 18m:  maternal age  married  white  private insurance  female  birth weight  singleton  SGA  prenatal care  prenatal steroid use  cesarean section  sepsis  intraventricular hemorrhage grade 3 or 4  periventricular leukomalacia  chronic lung disease  retinopathy of prematurity  necrotizing enterocolitis  duration of mechanical ventilation  time in NICU  postnatal steroid use  **validation**: not done | model 1:  mortality at 18m:  R^2^ = 0.4175  cluster infant factors present at birth:  R^2^ = 0.3700  model 2:  unimpaired or mildly impaired vs NDI or death at 18m:  R^2^= 0.2897  cluster infant factors present at birth  R^2^ = 0.2532  model 3:  unimpaired or mildly impaired vs NDI or death at 18m:  R^2^ = 0.2417  cluster infant factors present at birth  R^2^ = 0.0849  **range**: *not reported* | “Although <1% of live-born infants of <=500 g survive free of impairment at 18 months, this increases to almost 24% for infants of 901 to 1000 g. Female gender, singleton birth, higher birth weight, absence of neonatal morbidities, private health insurance, and white race increase the likelihood of unimpaired status.” |

1. Calculated from: Van Meurs KP, Wright LL, Ehrenkranz RA, Lemons JA, Ball MB, Poole WK, Perritt R, Higgins RD, Oh W, Hudak ML, Laptook AR, Shankaran S, Finer NN, Carlo WA, Kennedy KA, Fridriksson JH, Steinhorn RH, Sokol GM, Konduri GG, Aschner JL, Stoll BJ, D'Angio CT, Stevenson DK; Preemie Inhaled Nitric Oxide Study. Inhaled nitric oxide for premature infants with severe respiratory failure. N Engl J Med. 2005 Jul 7;353(1):13-22. [↑](#footnote-ref-2)
2. model available as an online calculator: http://www.nichd.nih.gov/about/org/cdbpm/pp/prog_epbo/epbo_case.cfm [↑](#footnote-ref-3)
