# Supplementary material for: Prediction of Mortality in Very Premature Infants: A Systematic Review of Prediction Models
Source: PLoS One. 2011 Sep 8;6(9):e23441. doi: 10.1371/journal.pone.0023441 (PMC3169543; doi:10.1371/journal.pone.0023441)
Supplement: Table S4 — Summary of input variables used in the 41 development studies. A total of 241 different input variables were used in the 41 studies describing development of a new prediction model. This table summarizes the variables by category. A complete list of the exact input variables used in each study, and their univariate and multivariate significance, is available from the authors upon request. (DOC) [file pone.0023441.s004.doc]

**Table S4: Summary of input variables used in the 41 development studies**

|  | size/maturity | | |  | | | maternal risk factors | | |  | | | | | | respiratory | | | | infant morbidities | | | |  | other | | |
| --- | --- | --- | --- | --- | --- | --- | --- | --- | --- | --- | --- | --- | --- | --- | --- | --- | --- | --- | --- | --- | --- | --- | --- | --- | --- | --- | --- |
|  | gestational age | birth weight | size for gestational age | gender | ethnicity | antenatal steroids | maternal age | hypertension | other MR | multiple gestation | presentation | mode of delivery | inborn/outborn | Apgar | temperature | blood gas | respiratory distress | ventilation / O_2_ supplementation | other R | congenital malformation | seizures | shock | other IM | infant laboratory |  | | |
| Behnke 1987 | S | U |  |  |  |  |  |  |  |  |  |  |  | S |  |  |  |  |  |  |  |  |  |  |  | | |
| Patterson 1988 | S | U | U | N |  |  |  |  | U | N | U | U |  | S | U |  |  |  | S |  |  |  |  |  | MR (pre-eclampsia^U^, PROM^U^, antepartum hemorrhage^U^), respiratory signs^S^, year of birth^NS^ | | |
| Horbar 1988 |  | S |  | S | S |  |  |  |  |  |  |  |  |  |  |  |  |  |  |  |  |  |  |  | center of birth^S^ | | |
| Ales 1988 | -- | S |  |  | -- | -- | -- |  | -- |  |  | -- |  | S |  |  |  |  |  |  |  |  |  |  | crown-heel length^U^, head circumference^U^, parity^--^, MR(obstetric risk^--^), substance abuse^--^, smoking^--^, maternal medical risk^--^, cause of VLBW^--^, physician status^--^ | | |
| Tarnow-Mordi 1990 | S | S |  |  |  |  |  |  |  |  |  |  |  |  |  | S | S |  | S |  |  |  |  |  | R (FiO_2_^S^, 7 other), hospital^S^, year^S^ | | |
| Horbar 1993 |  | S | S | S | S | U |  |  | U | U | U | U |  | S |  |  |  |  |  |  |  |  |  |  | prenatal care^U^, maternal complications^U^ | | |
| INN 1993 | S | S |  |  |  | U |  |  |  | -- |  | -- | -- | -- | -- | S |  | -- | S | S | -- |  | -- | -- | pre-eclampsia^--^, resuscitation^--^, blood pressure^--^, R (respiratory diagnosis^--^, FiO_2_^S^, air leak^--^, 2 other), IM (cerebral hemorrhage^--^) | | |
| Carter 1995 | S | S | S | U | N |  | U |  | U |  | N | U |  | S |  |  | S |  | U |  | S |  | N | N | prenatal ultrasound^S^, internal fetal monitoring^NS^, labor initiation^NS^. family density^S^, maternal education^NS^, MR (incompetent cervix^NS^, postpartum complications^U^, pre-eclampsia^U^, spontaneous labor^S^, PROM^NS^. vaginal bleeding^NS^, placental disease^NS^), fetal distress^NS^, interstitial emphysema^U^, IM (infection^NS^, IVH^NS^, hydrocephalus^NS^, NEC^NS^) | | |
| Roth 1995 |  | S |  | S | S |  |  |  |  |  |  |  |  |  |  |  |  |  |  |  |  |  |  |  | year of birth^S^ | | |
| Ballot 1996 | U | U |  |  |  | N |  |  |  |  | N | N | N | U |  | S |  |  | S | U |  |  |  |  | R (FiO_2_^S^), CRIB^U^ | | |
| Horbar 1997 |  | S | S | S | S | S |  |  |  | S |  | S | N | S |  |  |  |  |  | S |  |  |  |  | prenatal care^S^ | | |
| Maier 1997 | -- | S |  | -- |  |  |  |  |  |  |  |  | -- | S | -- | S | S | S |  |  |  |  |  | -- | resuscitation^--^ | | |
| Sulkes 1998 | S | S | S | N |  |  | S | S | S |  | N | U |  | S |  |  | U |  |  |  | S |  | U |  | preterm^NS^, parity^NS^, gravidity^NS^, MR (cervical suture^NS^, oliogohydroamniosis^NS^, diabetes^NS^, PROM^S^, placental disease^NS^) fetal distress^S^, apnea^U^, IM (sepsis^NS^, IVH^U^, PDA^U^) | | |
| Fowlie 1998 | U | U |  |  |  |  |  |  |  |  |  |  |  |  |  | N |  |  | N | N |  |  | S |  | R (FiO2^NS^), IM (NEC^S^), CRIB-72h^S^ | | |
| Zernikow 1998 | S | S | -- |  | -- |  |  |  |  | -- |  | -- | S | S | S | S |  |  |  | S |  |  | -- |  | MR (PROM-24h^--^), emergency delivery^S^, transport duration^--^, condition vitally endangered^S^, days intubated^--^, ventilated on transport^--^, BPD^--^, IM (IVH^--^, PVL^--^, survery required^--^), antibiotics^--^, transfustions^--^, year^--^ | | |
| Draper 1999 | S | S |  | S | S |  |  |  | S |  |  |  |  |  |  |  |  |  |  |  |  |  |  |  |  | | |
| Richardson 2001 |  | S | S | -- | -- |  |  |  |  | -- |  |  |  | S | S | S |  |  | S |  | S |  | S | -- | BP^S^, R (PO_2_/FiO_2_ ratio^S^, oxygenation index^--^), apnea^--^, respiratory signs^--^, stool guaiac^--^, IM (urine output^S^), heart rate^--^ | | |
| Janota 2001 |  |  |  |  |  |  |  |  |  |  |  |  |  |  |  |  |  |  |  |  |  |  |  |  | respiratory^U^, acid-base^U^, CNS^U^, GI^U^, cardiovascular^U^, renal^U^, coagulation^U^ | | |
| Gera 2001 | U | S | N |  |  |  |  |  | N |  |  |  |  | U | N | S |  | S | S | N | N | S |  | N | obstetric risks^NS^, maternal illness^NS^, resuscitation^U^, BP^NS^, R (need O_2_^NS^, O_2_ gradient^S^, apnea^NS^, resp rate^NS^), IM (urine output^NS^, ROP^NS^), CRIB^NS^ | | |
| Parry 2003 | S | S |  | S |  |  |  |  |  |  |  | -- |  |  | S | S |  |  |  |  |  |  |  |  | surfactant^--^ | | |
| Marshall 2005 | S | S | U | S |  | S | U |  |  | N |  |  |  | S |  |  |  |  |  | S |  |  |  |  |  | | |
| Evans 2007 | S |  | S | S | U^*^ | N | N | S | N | N | N | N | U | U |  |  |  |  |  |  |  |  |  |  | MR (previous perinatal death^NS^, previous preterm birth^NS^, labor^NS^, PROM^NS^, p-PROM^NS^, antepartum hemorrhage^NS^), fetal distress^U^ | | |
| Ambalavanan 2008 | -- | S |  | S | -- |  |  |  |  |  |  | -- | S |  |  |  |  |  | S |  | -- |  | -- |  | age at admission^S^, R (FiO_2_^S^, oxygenation index^S^, air leak^--^, pulm, hemorrahge^--^), mode of ventilation^--^, IM (GI bleed^--^, other bleed^--^, diagnosis^--^, indomethicin^--^, surfactant^--^, sedation^--^, response to iNO_2_^--^) | | |
| Basu 2008 | S | S | U |  |  | U | N | N | S |  |  |  |  | U | S |  | N |  |  |  |  | S |  | N | crown-heel length^U^, head circumference^U^, MR (gravidity^NS^, anemia^NS^, heart disease^NS^, vaginal leaking^NS^, bleeding^S^), fetal distress^U^, meconium^U^, birth asphyxia^U^, apnea^U^, IM (ischemic encephalopathy^U^, IVH^U^) | | |
| Almeida 2008 | S | U | U | N |  | U | N | S |  | U |  | U |  | S |  |  | S | N |  |  |  |  |  |  | prenatal care^NS^, maternal education^NS^, MR (diabetes^NS^, infection^NS^), resuscitation^U^, IM (sepsis^NS^, PDA^U^), surfactant -delivery^NS^, -72h^U^, SNAP^U^, hospital^S^ | | |
| Rosenberg 2008 | S | S |  | S |  |  |  |  |  |  |  |  |  |  | -- |  | N |  |  |  |  |  |  |  | birth asphyxia^NS^, cry at birth^NS^, apnea^NS^, grunting^NS^, tachypnea^NS^, lethargy^NS^, poor feeding^NS^ | | |
| Cole 2010 | S |  | S | S |  |  |  |  |  |  |  |  |  |  |  | S |  |  |  |  |  |  |  |  |  | | |
| Ballot 2010 |  | S | U | S |  | N |  |  |  |  | N | U | S | N | U |  | U | S |  |  |  |  | S |  | prenatal care^U^, resuscitation^S^, hypotension^S^, IM (sepsis^NS^, gram neg/pos^NS^, IVH/PVL^U^, NEC^S^, PDA^NS^, HIV^NS^, syphilis^NS^, surfactant^U^) | | |
|  | size/maturity | | |  | | | maternal risks factors | | |  | | | | | | respiratory | | | | infant morbidities | | | |  | other | | |
|  | gestational age | birth weight | size for gestational age | gender | ethnicity | antenatal steroids | maternal age | hypertension | other MR | multiple gestation | presentation | mode of delivery | inborn/outborn | Apgar | temperature | blood gas | respiratory distress | ventilation / O_2_ supplementation | other R | congenital malformation | seizures | shock | other IM | infant laboratory |  | | |
| Herschel 1982 | S | -- |  | N | N | S |  |  |  |  | N | N |  |  |  |  |  | N |  |  |  |  |  |  | hospitalization^NS^, MR (labor^NS^, PROM^NS^, abruptio placentae^NS^), resuscitation^S^, IM (sepsis^NS^) | | |
| Zarfin 1986 |  | S |  |  |  |  |  |  |  |  |  | N |  | U | S | S |  |  |  |  |  |  |  |  | fused eyes^NS^, MR (PROM^NS^, vaginal discharge^NS^), BP^U^, R (FiO2^S^, spontaneous breathing^S^, air leak^NS^, PIP^U^), time in NICU^U^, | | |
| Amon 1987 | S | S | N | S | S |  | N |  |  | N | N | N |  | S |  |  |  |  |  |  |  |  |  |  | prenatal care^NS^, maternal meds^NS^, anesthesia^NS^, cervical dilation^S^, time - admission to delivery^U^, MR (parity & gravidity^NS^, diagnosis^NS^), year^NS^ | | |
| Tyson 1996 | S | S | S |  | N | S |  |  |  | N |  |  |  | U |  |  |  |  |  |  |  |  |  |  | center of birth^NS^ | | |
| Doyle 2001 | S | U | S | S | N | S |  |  |  | S |  | N |  |  |  |  |  | N |  |  |  |  | U |  | BPD^NS^, IM (CVH^U^, PVL^NS^, surgery^U^, infant steroids^U^, surfactant^NS^) | | |
| Ambalavanan 2001 | S | S |  | -- | S | S |  | -- |  | -- | -- | -- | -- | S |  |  | S | -- |  |  |  |  |  |  | betamimetics^--^, Mg sulfate^--^, MR (p-PROM^--^, antepartum hemorrhage^--^, chorioamnionitis^--^), fetal distress^--^, year^--^ | | |
| Locatelli 2005 | S | S | N | S |  | S |  |  |  |  |  | N |  | S |  | N |  |  |  |  |  |  |  |  | MR (oligohydroamniosis^NS^, pre-eclampsia^NS^, PROM^NS^, placental disease^NS^), indicated delivery^U^ | | |
| Ambalavanan 2005 | S | S |  | S | S | S | -- | S | S | S |  |  |  | S |  |  | S | S | S |  |  |  | -- |  | prenatal care^--^, tocolytics^S^, antibiotics^S^, marital status^S^, MR (parity^--^, gravidity^--^, labor^--^, prepartum hemorrhage^S^), R (O_2_^S^, abnormal chest xray^--^), IM (indomethicin^--^), center mortality rate^S^ | | |
| Ambalavanan 2006 | S | S |  | -- | S | S | -- |  |  | -- |  | -- | -- | S |  |  |  | -- |  |  |  |  | S | S | tocolytics^--^, Mg sulfate^--^, family composition^--^, education^--^, MR (pre-eclampsia^--^, PROM^--^), air leak^--^, pulmonary hemorrahge^--^, IM (sepsis^--^, meningitis^--^, cranial ultrasound abnormalities^S^, bowel perforation^--^, PDA^--^, urine output^--^, transfusion^S^, crystalloids^S^) | | |
| Forsblad 2007 |  | N |  | N |  |  |  |  |  | S |  | N |  | S |  | N |  |  |  |  |  |  |  |  | CRIB^S^, level of care^NS^ | | |
| Tyson 2008 | S | S |  | S | -- | S |  |  |  | S |  | -- |  |  |  |  |  |  |  |  |  |  |  |  |  | | |
| Forsblad 2008 |  | U | U | N |  |  | N |  |  | S |  | N |  | S |  |  |  |  |  | N |  |  |  |  | level of care^NS^ | | |
| Gargus 2009 | U | S^†^ | S^†^ | S^†^ | S^†^ | S^†^ | S^†^ |  |  | S^†^ |  | S^†^ |  |  |  |  |  |  |  |  |  |  | N |  | birth length^U^, head circumference^U^, prenatal care^S†^, marital status^S†^, maternal education^NS^, insurance^NS^, duration of ventilation^U^, chronic lung disease^NS^, IM (sepsis^NS^, IVH^NS^, PVL^NS^, NEC^NS^, ROP^NS^), time to regain weight^U^, time in NICU^U^, number of morbidities^U^, infant steroids^NS^ | | |
| S – significant in multivariate analysis, included in final model  U – significant in univariate analysis, but not retained in final model  -- - univariate significance not reported, not retained in final model  N – not significant in univariate analysis and not retained in final model  ^*^variable omitted due to missing data  ^†^included in final model as part of a cluster  A complete list of input variables and their univariate and multivariate significance in each study is available upon request from the authors. | | | | | | | | | | | | | | | | | | | | | | | | | | PROM: premature rupture of membranes  p-PROM: prolonged premature rupture of membranes  FiO_2_: fraction inspired oxygen  IVH: intraventricular hemorrhage  NEC: necrotizing enterocolitis  PDA: patent ductus arteriosis  BPD: bronchopulmonary dysplasia  PVL: periventricular leukomalacia | BP: blood pressure  PO_2_: partial pressure of oxygen  CNS: central nervous system  GI: gastrointestinal (system)  ROP: retinopathy of prematurity  iNO_2_: inhaled nitric oxide  PIP: peak inspiratory pressure |

A total of 241 different input variables were used in the 41 studies describing development of a new prediction model. This table summarizes the variables by category. A complete list of the exact input variables used in each study, and their univariate and multivariate significance, is available from the authors upon request.
