# Supplementary material for: Prediction of Mortality in Very Premature Infants: A Systematic Review of Prediction Models
Source: PLoS One. 2011 Sep 8;6(9):e23441. doi: 10.1371/journal.pone.0023441 (PMC3169543; doi:10.1371/journal.pone.0023441)
Supplement: Text S1 — Framework for assessing quality of studies reporting the development of a prediction model, and scores of the 41 development studies. (DOC) [file pone.0023441.s005.doc]

**Text S5**: **Framework for assessing quality of studies reporting the development of a prediction model, and scores of the 41 development studies**

**Methodological score**:

1. n in development set is sufficient relative to the number of variables in the final model: [y: >10 events/variable -- partly: 5-10 events/variable -- n: <5 events/variable -- cannot determine]
   Depends on:
   1. n is reported: [y/partly/n]
      1. a. n in development set is reported as an n or a % [n can be approximated = partly]
      2. b. If there is a validation set, then the n in validation set is also reported as an n or a % [n reported for total population but not separately, or development but not validation, or vice versa, = partly]
   2. B. n with event is reported: [y/partly/n]
      1. a. n with event in development set is reported as an n or a %
      2. b. If there is a validation set, then the n with event in validation set is also reported as an n or a %
2. The population in the development set is representative of the population described by the aim or conclusions of the study. [y/partly - aim is not clear/n - inclusion not reported] 
   The limitations of the study should be acknowledged.  If inclusion/exclusion or handling of missing data excludes more than 10% of the general population, then the authors should acknowledge that the model may not apply to the excluded subpopulation. Note that including a variable with a high percentage of missing values in logistic regression is effectively the same as excluding this population from the model development process.
3. The model is validated [separate sample or bootstrap/partly or maybe/apparent - none - not reported]

**Reporting quality score**:

1. Description of study population
   1. setting and study period are described [y/partly/n]
   2. prospective/retrospective reported [y/partly/n]
   3. patient characteristics are described [y/partly/n]
      The development population is described in terms of the variables in the final model [only some of the variables in the final model are described = partly.  Unless split by randomization, the development and validation populations must be compared. If only the population as a whole is described = partly.]
2. Description of predictors/variables
   1. Choice of predictors/variables to test
      1. Initial predictors/variables are reported such that the results are reproducible [y/partly/n] [In most cases, simply listing the variables is sufficient, but the units of measure and the time of measurement should be given or be implicit from routine clinical practice.*]
      2. A reason is given for choosing these variables [y/partly/n]
      3. Initial variables include factors which are already known to be important, unless a reason for exclusion of these variables is given or implicit** [y - NA/partly/n]
      4. States or can be inferred which variables were continuous and which were categorical. If continuous predictors are converted to categorical then a reason is given or implicit*** [y - NA/partly/n - can't tell]
   2. Missing data
      1. Completeness of data is reported [y - NA/partly/n] [NA in situations where missing data is unlikely: if the model uses only a few variables and routinely collected data, then it is likely that missing data wasn't a problem and so the authors didn't mention it.]
      2. Handling of missing values is described [y - NA/partly/n] [This question addresses only whether it is described - if missing data was handled in such a way that it may compromise the integrity of the model, then a point should be deducted from part (3) of the methodological score above.]
   3. Outcome explicitly defined**** [y/partly/n]
3. Model description
   1. Intended use of model is described [y/partly/n]
   2. Model presentation
      1. The type of model is described (e.g. type of regression model) [y/partly/n or not described, model type _________________]
      2. Reporting of model derivation and calibration process is sufficient for the results to be reproduced [y/partly/n] [Report how variables were included/excluded in the final model and how the model was fitted to the data.]
      3. Model is presented such that it can be used to make a prediction [y/partly/n] [e.g. coefficients given in a regression model]
   3. 2 or more different measures of discrimination, calibration, or accuracy***** [y/partly/n] number ________
      1. spread reported for primary outcome measure(s) [y/partly/n] [e.g. CI or SE]
   4. Reports probabilities for at least two instances of the model (range or examples which imply range) [y/partly/n]
   5. Compares a performance measure of the new model with that of another model, using the same outcome and the same population.****** [y/partly/n]
   6. Reports individual contribution of most important single item in model [y/partly/n]

| * | In our review, we require: | |
| --- | --- | --- |
|  |  | units of birth weight: g vs 100g, 250g or 500g increments time of measurement for blood gasses and O2 requirement  unambiguous definitions of terms such as "obstetric risk" and "maternal complications" time of measurement of Apgar score |
| ** | In our review, we require only that they include either birth weight or gestational age in their initial variables, unless a reason is given or implicit. | |
| *** | In our review we accept that gestational age is usually recorded in completed weeks, thus no further explanation is needed. | |
| **** | In our review, outcome = mortality is part of the inclusion criteria, so we require that the outcome is further specified by the time of measurement: 7-day mortality is going to be quite different from 28-day mortality. | |
| ***** | In our review, measures included are: AUC, H-L, Brier score, R^2, contribution to variance, sensitivity, specificity, PPV, NPV, correct classification rate, accuracy. At least 1 measure of discrimination, calibration, or accuracy is in our study inclusion criteria. | |
| ****** | In our review, if there are multiple models reported, we require this from only one model. | |

| study | methodological score | events per variable | n | n-events | n-variables | inclusion representative | validated | setting | pros/retro | demographics | variables defined | reason for initial var. | important var. included | continuous-categorical | missing data reported | handling missing data | outcome | intended use | type | derivation | can use model | ≥2 performance measures | CI / SE | range or ≥2 instances of model | compare to other model | important var | reporting score |
| --- | --- | --- | --- | --- | --- | --- | --- | --- | --- | --- | --- | --- | --- | --- | --- | --- | --- | --- | --- | --- | --- | --- | --- | --- | --- | --- | --- |
| Behnke 1987 | 9 | Y | Y | Y | Y | P^*^ | N | Y | Y | Y | Y | Y | Y | N | N | N | Y | Y | Y | N | N | Y | N | N | Y | Y | 24 |
| Patterson 1988 | 12 | Y | Y | Y | Y | Y | Y | Y | N | Y | Y | N | Y | N | N | Y | Y | Y | Y | Y | N | N | N | Y | N | N | 20 |
| Horbar 1988 | 10 | Y | Y | Y | Y | Y | N | Y | Y | Y | Y | N | Y | N | P | P | Y | Y | Y | P | N | N | N | P | N | N | 20 |
| Ales 1988 | 12 | Y | Y | Y | Y | Y | Y | Y | Y | P | P | N | Y | N | P | P | Y | Y | Y | N | Y | Y | Y | Y | Y | Y | 28 |
| Tarnow-Mordi 1990 | 9 | N | Y | Y | Y | Y | P | Y | Y | Y | Y | Y | Y | Y | Y | Y | Y | Y | Y | Y | Y | Y | N | N | Y | Y | 34 |
| Horbar 1993 | 12 | Y | Y | Y | Y | Y | Y | Y | P | Y | Y | P | Y | P | Y | Y | Y | Y | Y | Y | Y | Y | Y | P | Y | Y | 34 |
| INN 1993 | 12 | Y | Y | Y | Y | Y | Y | Y | Y | N | Y | P | Y | Y | Y | Y | Y | Y | Y | Y | Y | Y | Y | Y | Y | Y | 35 |
| Carter 1995 | 7 | P | Y | Y | Y | N^†^ | N | Y | N | Y | P | Y | Y | Y | Y | Y | Y | Y | Y | P | Y | Y | N | P | N | Y | 29 |
| Roth 1996 | 8 | Y | P | P | Y | Y | N | Y | Y | Y | Y | N | Y | N | P | N | Y | Y | Y | Y | Y | N | N | Y | N | Y | 25 |
| Ballot 1996 | 10 | Y | Y | Y | Y | Y | N | Y | Y | Y | Y | Y | Y | Y | Y | Y | N | Y | Y | P | Y | N | N | N | N | Y | 27 |
| Horbar 1997 | 12 | Y | Y | Y | Y | Y | Y | Y | Y | Y | Y | Y | Y | P | P | Y | Y | Y | Y | Y | N | Y | Y | N | N | N | 28 |
| Maier 1997 | 11 | Y | Y | Y | Y | P^†^ | Y | Y | Y | Y | Y | Y | Y | N | P | Y | Y | Y | Y | Y | Y | Y | N | Y | Y | Y | 33 |
| Sulkes 1998 | 10 | Y | Y | Y | Y | Y | N | Y | Y | Y | Y | Y | Y | Y | N | N | Y | Y | Y | Y | Y | Y | Y | Y | N | Y | 32 |
| Fowlie 1998 | 9 | Y | Y | Y | Y | P^**^ | N | Y | Y | Y | Y | Y | Y | Y | Y | Y | Y | Y | Y | N | Y | Y | Y | N | Y | Y | 34 |
| Zernikow 1998 | 10 | N | Y | Y | Y | Y | Y | Y | Y | Y | Y | Y | Y | N | N | N | Y | Y | Y | Y | N | N | Y | Y | Y | Y | 28 |
| Draper 1999 | 10 | Y | Y | Y | Y | Y | N | Y | Y | N | Y | P | Y | Y | Y | Y | Y | Y | Y | Y | Y | N | N | Y | N | N | 27 |
| Richardson 2001 | 12 | Y | Y | Y | Y | Y | Y | Y | Y | P | Y | Y | Y | Y | P | Y | Y | Y | Y | Y | Y | Y | y | Y | N | Y | 34 |
| Janota 2001 | -- | NA | Y | Y | Y | P | N | Y | N | N | Y | Y | N | NA | N | Y | Y | Y | Y | NA | Y | P | N | Y | Y | NA | -- |
| Gera 2001 | 7 | P | Y | Y | P | P^**^ | N | Y | Y | Y | Y | N | Y | Y | N | N | N | Y | Y | Y | P | N | N | N | Y | Y | 23 |
| Parry 2003 | 11 | Y | Y | P | Y | Y | Y | Y | Y | P | Y | Y | Y | Y | N | N | Y | Y | Y | Y | Y | Y | Y | Y | Y | Y | 33 |
| Marshall 2005 | 12 | Y | Y | Y | Y | Y | Y | Y | Y | P | Y | Y | Y | Y | N | N | Y | Y | Y | Y | Y | Y | N | P | Y | Y | 30 |
| Evans 2007 | 12 | Y | Y | Y | Y | Y | Y | Y | Y | Y | Y | P | Y | N | Y | Y | Y | Y | Y | Y | Y | Y | N | N | Y | Y | 31 |
| Ambalavanan 2008 | 10 | Y | Y | Y | Y | Y | N | Y | Y | P | Y | Y | Y | N | P | P | Y | Y | Y | P | P | P | N | P | Y | Y | 27 |
| Basu 2008 | 10 | Y | Y | Y | Y | Y | N | Y | Y | Y | Y | P | Y | Y | N | N | Y | P | Y | N | Y | N | N | N | N | Y | 22 |
| Almeida 2008 | 10 | Y | Y | Y | Y | Y | N | Y | Y | Y | Y | N | Y | N | P | N | Y | Y | Y | Y | N | N | N | N | N | Y | 21 |
| Rosenberg 2008 | 11 | Y | Y | Y | Y | P^‡^ | Y | Y | Y | Y | Y | Y | Y | Y | Y | Y | Y | Y | Y | Y | Y | Y | Y | Y | Y | Y | 38 |
| Cole 2010 | 11 | Y | Y | Y | Y | Y | P | Y | P | Y | Y | Y | Y | Y | Y | Y | Y | Y | Y | Y | Y | Y | N | Y | Y | Y | 35 |
| Ballot 2010 | 10 | Y | Y | Y | Y | Y | N | Y | Y | Y | Y | P | Y | N | Y | Y | Y | Y | Y | Y | N | N | N | N | Y | Y | 27 |
| study | methodological score | events per variable | n | n-events | n-variables | inclusion representative | validated | setting | pros/retro | demographics | variables defined | reason for initial var. | important var. included | continuous-categorical | missing data reported | handling missing data | outcome | intended use | type | derivation | can use model | ≥2 performance measures | CI / SE | range or ≥2 instances of model | compare to other model | important var | reporting score |
| Herschel 1982 | 10 | Y | Y | Y | Y | Y | N | Y | N | Y | P | N | Y | N | N | N | Y | N | Y | N | N | N | N | N | N | N | 11 |
| Zarfin 1986 | 10 | Y | Y | Y | Y | Y | N | Y | Y | P | Y | N | Y | Y | N | N | P | N | Y | Y | N | Y | N | N | Y | Y | 22 |
| Amon 1987 | 9 | Y | Y | Y | Y | P^**^ | N | Y | Y | Y | Y | N | Y | Y | N | N | Y | Y | Y | Y | N | N | N | N | N | N | 20 |
| Tyson 1996 | 12 | Y | Y | Y | Y | Y | Y | Y | Y | Y | Y | Y | Y | Y | Y | Y | Y | Y | Y | P | P | Y | N | Y | N | Y | 32 |
| Doyle 2001 | 10 | Y | Y | Y | Y | Y | N | Y | Y | Y | N | Y | Y | Y | P | P | Y | Y | Y | N | N | N | N | Y | N | N | 22 |
| Ambalavanan 2001 | 11 | P | Y | Y | Y | Y | Y | Y | Y | Y | Y | Y | Y | Y | N | N | N | Y | Y | P | P | Y | Y | N | Y | Y | 28 |
| Locatelli 2005 | 9 | P | Y | Y | Y | Y | N | Y | Y | Y | Y | P | Y | P | Y | Y | Y | Y | Y | Y | N | Y | N | N | N | Y | 28 |
| Ambalavanan 2005 | 12 | Y | Y | Y | Y | Y | Y | Y | Y | P | Y | Y | Y | P | P | Y | P | Y | Y | Y | P | Y | Y | N | Y | Y | 31 |
| Ambalavanan 2006 | 12 | Y | Y | Y | Y | Y | Y | Y | Y | P | Y | Y | Y | Y | Y | Y | Y | Y | Y | Y | Y | Y | Y | Y | Y | Y | 37 |
| Forsblad 2007 | 9 | Y | Y | Y | Y | P^†^ | N | Y | Y | Y | Y | P | Y | N | Y | Y | Y | Y | Y | P | Y | N | N | Y | Y | Y | 30 |
| Tyson 2008 | 12 | Y | Y | Y | Y | Y | Y | Y | Y | Y | Y | Y | Y | Y | P | Y | P | Y | Y | Y | Y | N | Y | Y | Y | Y | 34 |
| Forsblad 2008 | 9 | Y | Y | Y | Y | P^†^ | N | Y | Y | Y | Y | P | Y | Y | Y | Y | Y | Y | Y | P | Y | N | N | Y | N | Y | 30 |
| Gargus 2009 | 10 | Y | Y | Y | Y | Y | N | Y | Y | Y | Y | N | Y | P | Y | Y | Y | Y | Y | Y | N | N | N | N | Y | Y | 27 |
| **totals** |  | 34 | 40 | 39 | 40 | 31 | 17 | 40 | 35 | 30 | 36 | 21 | 40 | 22 | 17 | 23 | 34 | 37 | 40 | 26 | 22 | 22 | 14 | 18 | 23 | 33 |  |

^*^ Inclusion criteria not reported

^†^ Excluded >10% due to missing data (Carter: 30%, Maier: 11.5%, Forsblad 2007: 25% for one model and 4% for others, Forsblad 2008: 10% for one model) without comparison of characteristics of included vs. excluded infants or sensitivity analysis.

^**^ Excluded outborn infants, although the center is a referral center or has a large outborn population, without an assessment of whether the inborn population is representative.

^‡^ Excluded infants “judged unlikely to live beyond 48 hours”
