# Supplementary figures and images for: Prediction of Mortality in Very Premature Infants: A Systematic Review of Prediction Models
Source: PLoS One. 2011 Sep 8;6(9):e23441. doi: 10.1371/journal.pone.0023441 (PMC3169543; doi:10.1371/journal.pone.0023441)

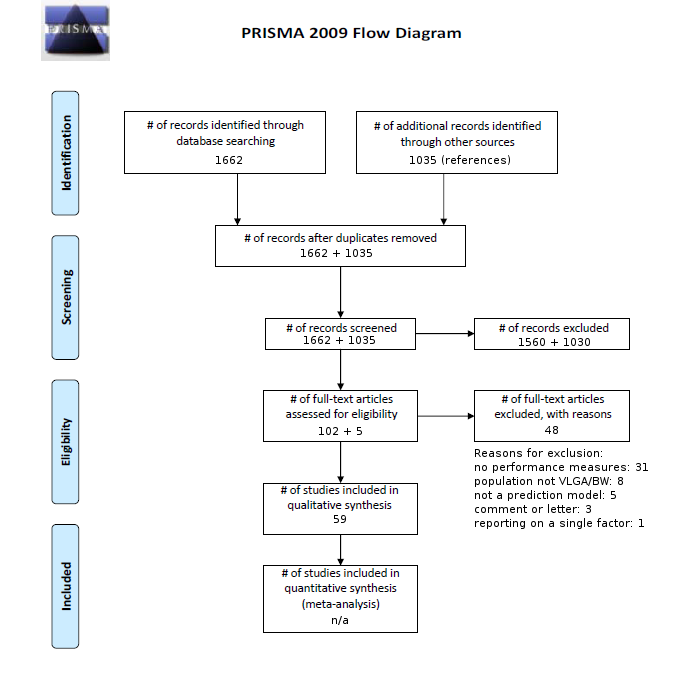

Supplement: Diagram S1 — PRISMA flow diagram. (TIF) [file pone.0023441.s007.tif]
